# Collapse of feed-forward inhibition underpins hyperexcitability in GABA_A_ receptor gain-of-function epilepsy

**DOI:** 10.1093/brain/awag119

**Published:** 2026-04-22

**Authors:** Chaseley E McKenzie, Khaing Phyu Aung, Altair Brito Dos Santos, Miaomiao Mao, James Spyrou, Alexander Bryson, Raymond Wong, Susan X N Lin, Lauren E Bleakley, Joakim Palmqvist, Robertas Guzulaitis, Anthony S H Kan, Vivian W Y Liao, Ming S Soh, Carlos Daniel Gomez, Victor Larsen, Anne Marie Nordvig Petersen, Nathan L Absalom, Rikke S Møller, Mary Chebib, Jean-François Perrier, Philip K Ahring, Christopher A Reid

**Affiliations:** The Florey Institute of Neuroscience and Mental Health, University of Melbourne, Parkville, Victoria 3010, Australia; The Florey Institute of Neuroscience and Mental Health, University of Melbourne, Parkville, Victoria 3010, Australia; Department of Neuroscience, Faculty of Health and Medical Sciences, University of Copenhagen, Copenhagen 2200, Denmark; Brain and Mind Centre, School of Medical Sciences, Faculty of Medicine and Health, The University of Sydney, Sydney, New South Wales 2006, Australia; The Florey Institute of Neuroscience and Mental Health, University of Melbourne, Parkville, Victoria 3010, Australia; The Florey Institute of Neuroscience and Mental Health, University of Melbourne, Parkville, Victoria 3010, Australia; Department of Neurology, Eastern Health, Victoria 3128, Australia; Centre for Eye Research Australia, University of Melbourne, Victoria 3002, Australia; Brain and Mind Centre, School of Medical Sciences, Faculty of Medicine and Health, The University of Sydney, Sydney, New South Wales 2006, Australia; The Florey Institute of Neuroscience and Mental Health, University of Melbourne, Parkville, Victoria 3010, Australia; Department of Neuroscience, Faculty of Health and Medical Sciences, University of Copenhagen, Copenhagen 2200, Denmark; Life Sciences Center, Vilnius University, Vilnius LT-10257, Lithuania; Brain and Mind Centre, School of Medical Sciences, Faculty of Medicine and Health, The University of Sydney, Sydney, New South Wales 2006, Australia; Brain and Mind Centre, School of Medical Sciences, Faculty of Medicine and Health, The University of Sydney, Sydney, New South Wales 2006, Australia; The Florey Institute of Neuroscience and Mental Health, University of Melbourne, Parkville, Victoria 3010, Australia; Department of Neuroscience, Faculty of Health and Medical Sciences, University of Copenhagen, Copenhagen 2200, Denmark; Department of Neuroscience, Faculty of Health and Medical Sciences, University of Copenhagen, Copenhagen 2200, Denmark; Department of Neuroscience, Faculty of Health and Medical Sciences, University of Copenhagen, Copenhagen 2200, Denmark; Brain and Mind Centre, School of Medical Sciences, Faculty of Medicine and Health, The University of Sydney, Sydney, New South Wales 2006, Australia; School of Science, University of Western Sydney, Sydney, New South Wales 2751, Australia; Department of Epilepsy, Genetics and Personalized Medicine, Member of ERN, EpiCare, Danish Epilepsy Centre, Dianalund DK-4293, Denmark; Department of Regional Health Research, Faculty of Health Sciences, University of Southern Denmark, Odense DK-5230, Denmark; Brain and Mind Centre, School of Medical Sciences, Faculty of Medicine and Health, The University of Sydney, Sydney, New South Wales 2006, Australia; Department of Neuroscience, Faculty of Health and Medical Sciences, University of Copenhagen, Copenhagen 2200, Denmark; Brain and Mind Centre, School of Medical Sciences, Faculty of Medicine and Health, The University of Sydney, Sydney, New South Wales 2006, Australia; The Florey Institute of Neuroscience and Mental Health, University of Melbourne, Parkville, Victoria 3010, Australia

**Keywords:** hypoactivity, *GABRB3*, synaptic function, hippocampus, cortex, developmental and epileptic encephalopathy

## Abstract

Gain-of-function (GOF) variants in γ-aminobutyric acid type A (GABA_A_) receptors are increasingly recognized as a cause of severe developmental and epileptic encephalopathies. However, the mechanisms by which enhanced GABA_A_ receptor activity leads to neuronal network hyperexcitability remain unclear. We engineered a new mouse model based on the human GOF *GABRB3* p.(Glu77Lys) variant (β3^E77K^), identified in two individuals diagnosed with developmental and epileptic encephalopathies, to explore the mechanisms underlying GABA_A_ receptor GOF disease. The phenotypes of β3^E77K^ mice included embryonic lethality consistent with a severe early developmental impact of the GOF GABA_A_ receptor variant. These mice display spike-wave-like discharges that were exacerbated by vigabatrin and ameliorated by valproate, matching the clinical observations of GOF *GABRB3* patients. β3^E77K^ mice also have increased proconvulsant-induced seizure susceptibility and a broad increase in electrocorticographic spectral power amplitude, indicative of cortical hyperexcitability. Additionally, neurological assessments revealed hypoactivity and weakened grip strength.

*Ex vivo* electrophysiological recordings demonstrated increased GABA_A_ receptor-mediated current amplitudes at both excitatory and inhibitory synapses in CA1 hippocampus, consistent with the GOF molecular phenotype previously identified in functional studies. In cortical layer 2/3, inhibitory interneurons showed increased synaptic GABA_A_ receptor-mediated current amplitude, and synapses onto pyramidal neurons exhibited reduced inhibitory currents. Enhanced GABA_A_ receptor-mediated synaptic activity among layer 2/3 interneuron populations caused a use-dependent collapse of feed-forward inhibition, resulting in increased pyramidal neuron excitability. Computational modelling supported this disinhibition mechanism, showing that enhanced GABA_A_ receptor synaptic strength between interneurons diminishes inhibitory synaptic conductance onto pyramidal cells.

Our findings highlight the critical role of interneuron network dysfunction in driving cortical hyperexcitability caused by GOF GABA_A_ receptor variants. They provide a novel pathogenic mechanism in developmental and epileptic encephalopathies that could have broader implications for disorders involving dysfunction in GABAergic neurons. The β3^E77K^ mouse also provides a unique preclinical model to test therapeutic strategies in GOF GABA_A_ receptor disease.

## Teaser

Paradoxically, mutations that enhance GABA_A_ receptor function cause severe epilepsy. Using a mouse model of a human gain-of-function GABA_A_ receptor mutation, McKenzie *et al.* show that increased channel function causes excessive inhibition between inhibitory neurons, weakening feed-forward inhibition and increasing cortical excitability.

## Introduction

Ligand-gated ionotropic γ-aminobutyric acid type A (GABA_A_) receptors are pivotal in neuronal inhibition and in balancing the excitation–inhibition dynamics within the mammalian brain.^1^ Unsurprisingly, dysfunction in these receptors is strongly implicated in the pathophysiology of epilepsy, with variants in genes encoding GABA_A_ receptor subunits frequently found in patients with epilepsy.^2-10^ For >15 years, loss-of-function (LOF) of the GABA_A_ receptor has been reported as the primary mechanism linking GABA_A_ receptor variants to a spectrum of epilepsies, including the most severe developmental and epileptic encephalopathies (DEEs).^5^ However, LOF alone cannot account for the wide range of clinical phenotypes observed, nor does it adequately stratify patients according to the impact of the variant on GABA_A_ receptor function.

Recently, we and others have demonstrated that approximately half of known *GABRB3* pathogenic variants paradoxically result in gain-of-function (GOF) of the GABA_A_ receptor.^2,4,11,12^ GOF pathogenic variants are generally associated with more severe clinical phenotypes. For example, GOF variants in *GABRB3*, which encodes the β3 subunit of the GABA_A_ receptor, are associated with an earlier onset of seizures, more pronounced developmental delay and other distinct comorbidities in comparison to LOF variants.^2^ This indicates that distinct cellular and neuronal network-scale pathological mechanisms must underlie disease in the GOF and LOF patient groups.

One powerful method for assessing the impact of genetic pathogenic variants is through the creation of genetically engineered mice based on human pathogenic variants.^13^ Although this approach has been applied to epilepsy caused by GABA_A_ receptor variants, published mouse models to date appear to be based on variants with a LOF biophysical phenotype.^14-16^ An intuitive and simple pathological mechanistic basis of disease has emerged from *Gabrb3* LOF mouse models, which includes a reduction in synaptic inhibition, disrupted excitation–inhibition balance and resultant network-scale hyperexcitability.^2^ However, the strong association of GOF pathogenic variants with epilepsy and DEE cannot be explained by this simple mechanistic model. To address this conundrum, we engineered a mouse model based on the human *GABRB3* p.(Glu77Lys) (β3^E77K^) variant. This variant was identified in two individuals diagnosed with DEE, with functional analysis revealing increased GABA sensitivity, representing a GOF effect.^11^ Heterozygous *Gabrb3*^E77K^ (β3^E77K^) mice therefore represent an ideal model to investigate the mechanistic basis of GOF GABA_A_ receptor disease.

Heterozygous β3^E77K^ mice recapitulate several clinical phenotypes of *GABRB3* GOF disease, including spike-wave-like discharges on electrocorticography (ECoG), increased proconvulsant-induced seizure susceptibility, hypo-locomotion and impaired grip strength. Importantly, inhibitory current amplitudes are uniformly increased at hippocampal synapses and selectively increased at cortical inhibitory synapses, consistent with the GOF GABA_A_ receptor biophysical predictions from *in vitro* expression systems.^2,11,17^ Furthermore, cortical network-scale analysis revealed a ‘use-dependent’ reduction in layer 2/3 feed-forward inhibition that arises from enhanced inhibitory-to-inhibitory synaptic transmission. This mechanism of disinhibition is likely to underlie the increase in cortical excitability observed in the β3^E77K^ mouse. Hence, this study reveals a mechanism for how GOF variants cause epilepsy through experimental evidence.

## Materials and methods

### Animals and ethics approvals

All human and animal procedures complied with national and institutional regulations.

#### Ethics

This study includes previously published clinical data from individuals harbouring *GABRB3* variants, with informed consent obtained in the original studies. Ethics approval was granted by the Institutional Review Board at the Danish Epilepsy Centre (EMN-2024-01998).^11,18,19^

Oocyte extraction from *Xenopus laevis* frogs was performed following a protocol approved by the Animal Ethics Committee of The University of Sydney (AEC No. 2016/970) in accordance with the National Health and Medical Research Council of Australia code for the care and use of animals.

Mouse experiments performed at the University of Copenhagen (clasping and feed-forward inhibition assays) followed Danish animal welfare legislation and were approved by the Danish Animal Experiments Inspectorate (2018-15-0202-00157).

All other mouse experiments were conducted at the Florey Institute of Neuroscience and Mental Health (Australia) under approvals issued by the Florey Animal Ethics Committee, in accordance with the Prevention of Cruelty to Animals Act 1986 and the NHMRC Code. Mice were monitored daily, acclimated to procedure rooms for ≥1 h prior to experiments, and anaesthesia and analgesia were provided where appropriate. Humane killing was performed under deep anaesthesia using cervical dislocation or decapitation [Australian and New Zealand Council for the Care of Animals in Research and Training (ANZCCART)-approved methods].

#### Generation of the β3^E77K^ and other Gabrb3 mouse models

Knock-in mouse models were generated using CRISPR-based technologies on a C57Bl/6J background by the Monash Gene Modification Platform (MGMP) (Supplementary Fig. 1 and Supplementary Tables 1 and 2). For generation of β3^E77K^-6N mice, the established C57Bl/6J line was backcrossed for at least one generation onto the C57Bl/6N background. Full details of the generation of these mouse models can be found in the Supplementary material.

#### Animal housing and husbandry

Mice were group-housed under a 12 h–12 h light–dark cycle, with *ad libitum* access to food and water. β3^E77K^ mice were maintained on both C57Bl/6J and C57Bl/6N genetic backgrounds. All animals were PCR-genotyped before and/or after experiments.

At the Florey Institute of Neuroscience and Mental Health, experiments were conducted in two β3^E77K^ cohorts on controlled C57Bl/6 backgrounds. The primary experimental cohort consisted of heterozygous β3^E77K^ mice maintained on a C57Bl/6J background, bred with wild-type (WT) C57Bl/6J mice to generate β3^E77K^ and WT littermates. To assess reproducibility across genetic background, a subset of electrophysiological and behavioural studies was additionally performed in F1 hybrid mice generated by crossing heterozygous β3^E77K^ C57Bl/6J males with WT C57Bl/6N females. Littermates were used wherever possible.

At the University of Copenhagen, β3^E77K^ mice were maintained on a C57Bl/6N background, and breeding to WT C57Bl/6N mice produced β3^E77K^ and WT littermates for experimentation. F1 hybrid mice were also used in selected studies to evaluate whether phenotypes observed in C57Bl/6J mice were conserved across backgrounds.

#### Animal strains, sex and age

The principal phenotypic characterization of the β3^E77K^ variant was conducted in C57Bl/6J mice at the Florey Institute. Selected experiments were replicated in F1 C57Bl/6J × C57Bl/6N hybrid mice at both sites, and in C57Bl/6N mice at the University of Copenhagen. Both males and females were used for all experiments. Sample sizes for each cohort are provided in the corresponding figure legends. Mendelian segregation was analysed using χ^2^ tests.

### Transcardial perfusions and immunohistochemistry

Mice (aged 12–20 weeks) were anaesthetized and perfused with 4% paraformaldehyde in PBS. Brains were extracted and post-fixed overnight in 4% paraformaldehyde in PBS, then washed with PBS and stored in PBS at 4°C. Coronal brain sections (50 µm thick) were cut with a cryostat, and free-floating sections were used for immunofluorescence analysis. Details of the tissue preparation, staining protocols and analysis, can be found in the Supplementary material, ‘Methods’ section.

### Electrocorticography

#### Electrocorticography electrode implantation surgery

Male and female β3^E77K^ mice and WT littermates, 6–16 weeks of age, were used for electrocorticography (ECoG) experiments. Details of the ECoG electrode implantation surgery can be found in the Supplementary material, ‘Methods’ section.

#### ECoG recordings

Brain cortical activity was sampled at 250 Hz (Pinnacle Technology), with a low-pass and high-pass filter of 40 and 0.5 Hz, respectively. Twenty-four-hour recordings were conducted to characterize baseline ECoG activity during the light and dark phases. Aberrant rhythmic discharges were identified as spike-wave-like discharges comprising three or more consecutive spike–wave cycles at 7–10 Hz, lasting 0.3–2.0 s, visible across both cortical channels. Details of the ECoG recordings and analysis can be found in the Supplementary material, ‘Methods’ section.

#### Drug testing experiments

Additional ECoG experiments were conducted to assess the effect of anticonvulsant drugs on spike-wave-like activity following intraperitoneal injection of vigabatrin (300 mg kg^−1^), valproate (200 mg kg^−1^) or vehicle (saline); analysis details are provided in the Supplementary material, ‘Methods’ section.

### Subcutaneous pentylenetetrazole assay

Mice (aged 12–20 weeks) were injected subcutaneously with 100 mg kg^−1^ pentylenetetrazole (PTZ) and monitored for a maximum of 30 min. The time to first tonic–clonic seizure and time to hindlimb extension were recorded. Mice were culled by cervical dislocation after 30 min.

### Behavioural assays

Mice were allowed to acclimate in their home cages to the behavioural room for ≥1 h prior to the commencement of any behavioural assays. Mice were assessed in the open-field locomotor and thigmotaxis, light–dark box, rotarod, ledged beam, grip strength, clasping, elevated plus maze, Y-maze, three-chamber social interaction and Barnes maze assays. Details of each of these assays can be found in the Supplementary material, ‘Methods’ section.

### Two-electrode voltage-clamp experiments

Two-electrode voltage-clamp electrophysiology was performed using *Xenopus laevis* oocytes, as described previously.^11^ Details of the two-electrode voltage-clamp experiments and analysis can be found in the Supplementary material, ‘Methods’ section.

### Brain slice electrophysiology

For whole-cell recordings, brain slices were prepared from postnatal Day (P)20–P34 mice, and 6- to 12-week-old mice were used for feed-forward inhibition experiments. Details of the slice preparation, electrophysiological recordings and analysis for whole-cell recordings and feed-forward inhibition experiments can be found in the Supplementary material, ‘Methods’ section.

#### Layer 2/3 interneuron identification

Recorded layer (L)2/3 interneurons were identified using established physiological characteristics, including laminar location, morphology and low somatic membrane capacitance (<80 pF), with fast-spiking firing properties (firing frequency >80 Hz) assessed where applicable. Neurons were filled with biocytin during whole-cell recordings. In a subset, they were processed for *post hoc* immunohistochemistry to confirm parvalbumin (PV) expression. Immunostaining of biocytin-filled neurons and PV^+^ interneurons was performed as described previously.^20^ PV-verified interneurons for miniature inhibitory postsynaptic current (mIPSCs) recordings exhibited morphological and electrophysiological properties [membrane capacitance (in picofarads) = non-verified versus verified, *P* = 0.53, one-way ANOVA] comparable to those of the physiologically identified interneuron population, and data were therefore pooled (Supplementary Table 3). Full procedural details are provided in the Supplementary material, ‘Methods’ section.

### RNA-sequencing dataset

The mouse whole cortex and hippocampus single-cell RNA-sequencing dataset^21^ was available from the Allen Brain Map portal (https://portal.brain-map.org/atlases-and-data/rnaseq/mouse-whole-cortex-and-hippocampus-smart-seq). The dataset, metadata and processed *t*-distributed stochastic neighbour embedding (tSNE) coordinates were imported into *Seurat* (v.4.3) for analysis. The primary somatosensory region (SSp) was subset using the *subset* function, followed by data normalization by variance stabilizing transformation using *SCTransform* (v.2). Details of the annotation and visualization of gene expression can be found in the Supplementary material, ‘Methods’ section.

### Feed-forward network model

To examine the impact of GABA_A_ receptor GOF on cortical feed-forward inhibition, we created a network model containing an excitatory pyramidal cell (PC) and 50 inhibitory basket cells (BC). Details of the modelling can be found in the Supplementary material, ‘Methods’ section.

### Statistical analysis

All statistical analyses were done using GraphPad Prism (v.10.1.1, GraphPad Software). The normal distribution of the data was tested using the Anderson–Darling, D’Agostino and Pearson, Shapiro–Wilk and Kolmogorov–Smirnov tests. Parametric statistical tests were used for data that passed all these tests; otherwise, non-parametric tests were used (e.g. Mann–Whitney U-test). For normal data, the cells were compared using Student’s unpaired *t*-test. For multiple comparisons, two-way ANOVA was used. Results are given as the mean ± standard deviation (SD) or standard error of the mean (SEM), together with the *P*-value and *n*, representing the number of recorded cells. Significance was defined as *P* < 0.05. The significance and *P*-values are indicated in the figures.

### Exclusion criteria

For slice electrophysiology data, cells were excluded from analysis if access resistance was >30 MΩ at any time point or if it fluctuated by >20% during the recording session. Cells were also excluded from analysis if baseline voltage ran down significantly during the recording.

For mouse behavioural and EEG experiments, data >2SD away from the mean were excluded.

## Results

### Many *GABRB3* GOF variants are lethal in C57Bl/6J mice

In our attempt to model GOF *GABRB3* disease, we engineered four C57Bl/6J mouse models based on pathogenic variants from different functional regions of the β3 subunit, each with severe clinical phenotypes.^3,11^ The variants, in ascending order of observed clinical severity based both on the age of onset and combined clinical symptoms, included p.(Glu77Lys), p.(Ile306Thr), p.(Thr287Ile) and p.(Leu256Gln). Although founder mice were confirmed to carry the p.(Ile306Thr), p.(Thr287Ile) and p.(Leu256Gln) point mutations, repeated breeding attempts across multiple litters failed to produce viable heterozygous offspring, with variant-positive pups dying perinatally or before weaning, with some displaying severe seizures (Supplementary Table 2). As a result, stable breeding colonies could not be established for these lines, consistent with the severe consequences of GOF variants. Only the mouse model based on the p.(Glu77Lys) variant (*Gabrb3*^E77K^) produced viable pups and was thus used as a model for *GABRB3* GOF DEE. Both C57Bl/6J (herein β3^E77K^-6J) and backcrossed F1 C57Bl/6N (herein β3^E77K^-6N) genetic backgrounds were used in this study.

### Individuals carrying the *GABRB3* E77K variant have DEE

The p.(Glu77Lys) variant has been observed in two individuals. The first individual was a 5-year-old girl with severe developmental delay, language delay (few words at 5 years), limited social interaction, autism spectrum disorder, hyperactivity and hypotonia, who presented with epileptic spasms at 4 months of age. At 18 months of age, she became seizure free except for infrequent myoclonic seizures. Her EEG at presentation showed high-amplitude spike-and-wave and polyspike-and-wave discharges in the posterior region.^2,11,19^ The second individual was a female with moderate intellectual disability, dystonia, ataxia and spasticity, who presented with myoclonic and bilateral tonic–clonic seizures at 16 years of age. Her EEG showed generalized spike-and-wave discharges.^18^

### The *GABRB3* E77K GABA_A_ receptor displays a gain-of-function molecular phenotype

The p.(Glu77Lys) variant is located in loop 2 of the β3 subunit, within a structural area known as the coupling region (Fig. 1A). This region is important for transducing allosteric events from GABA binding in the extracellular domain to the opening of the channel in the transmembrane domain.^22^

**Figure 1:**
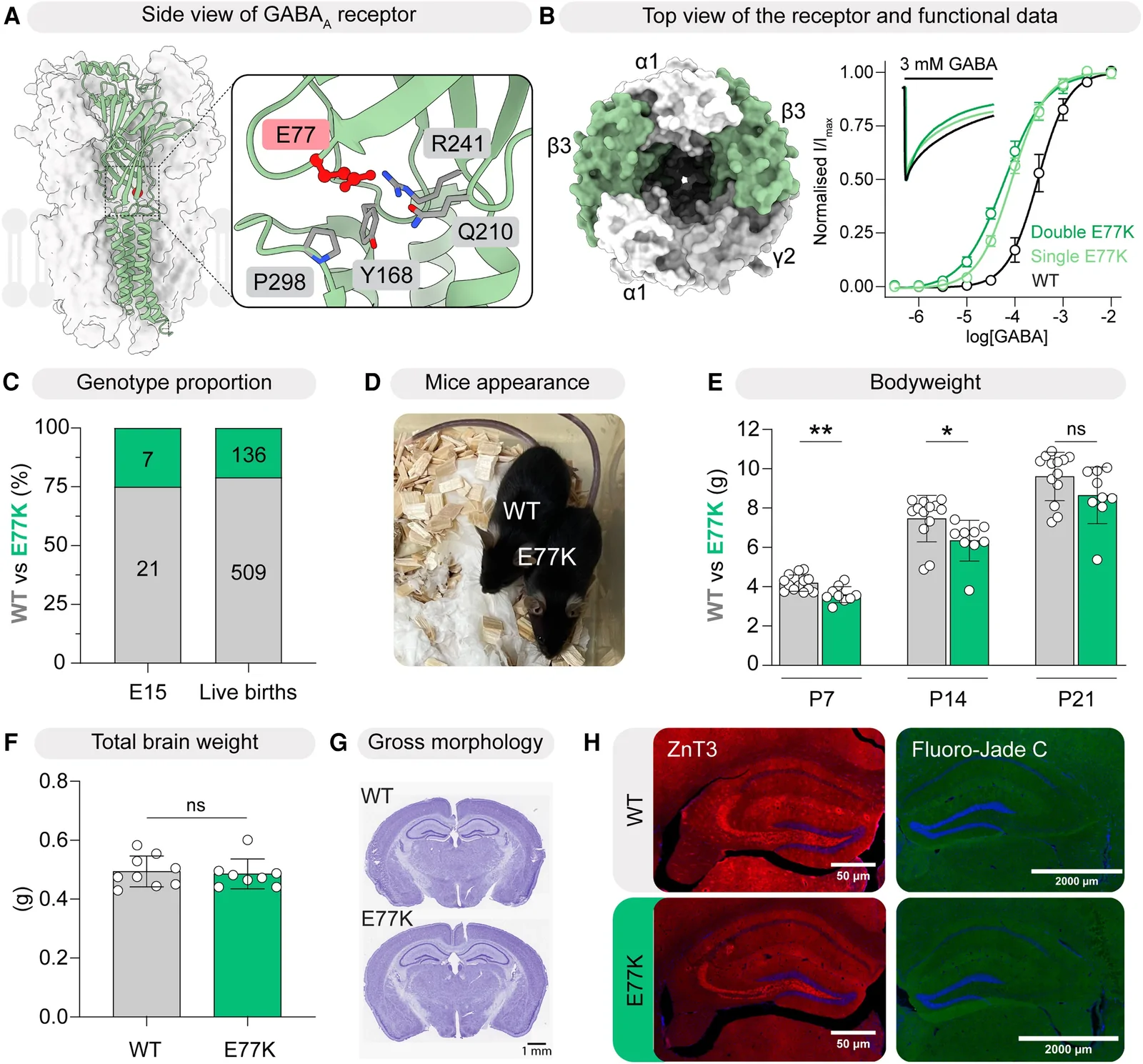
**Disease phenotype of β3^E77K^-6J mice.** (**A**) Cryo-electron microscopic structure of an α1β3γ2 GABA_A_ receptor, with β3 subunits highlighted in green (PDB: 6HUP). E77 is located in loop 2, near the base of the transmembrane domain. (**B**) Concentration–response relationship obtained from two-electrode voltage-clamp recordings demonstrates a gain in GABA sensitivity in receptors carrying the E77K variant in either one (ΔlogEC_50_ = 0.54 ± 0.06, *n* = 11, *P* < 0.0001) or both (ΔlogEC_50_ = 0.69 ± 0.082, *n* = 12, *P* < 0.0001) β3 subunits. The variant also led to increased desensitization rate in the double E77K variant receptor (*k* = 0.023 ± 0.0050 s^−1^, *n* = 14, *P* < 0.0001), as shown in the *inset* current traces. (**C**) Genotype proportions of wild-type (WT) and β3^E77K^ mice at embryonic Day 15 (E15; *n* = 28, **P = 0.0082, χ^2^ test) and at birth (*n* = 645, ****P < 0.0001, χ^2^ test). (**D**) β3^E77K^ mice (*right*) did not differ significantly in appearance compared with their WT littermates (*left*). (**E**) Body weight, measured at postnatal Day (P)7, P14 and P21 for β3^E77K^ mice and WT littermates (*n* = 13 WT, 9 β3^E77K^), showed a significant main effect of genotype (two-way repeated-measures ANOVA; *P* = 0.032), with no significant Genotype × Age interaction, indicating that β3^E77K^ mice were consistently lighter across ages (*n* = 13 WT, *n* = 9 β3^E77K^). (**F**) Brain weight was not significantly different between the genotypes (*n* = 9 WT, *n* = 8 β3^E77K^), *P* = 0.9999, Student’s unpaired *t*-test. (**G**) Gross morphology of whole brain slice from WT (*top*) and β3^E77K^ mouse (*bottom*). (**H**) Representative images of ZnT3 and Fluoro-Jade C immunostaining in the hippocampus showed no significant changes in mossy fibre sprouting and neuronal degeneration, respectively, between WT and β3^E77K^ brain slices. Data for **B**, **E** and **F** are presented as the mean ± standard deviation. *P < 0.05, **P < 0.01.

Two-electrode voltage-clamp recordings were conducted to assess functional alterations in WT and β3^E77K^-containing α1β3γ2 GABA_A_ receptors. In this concatenated receptor configuration, ‘single-variant’ receptors contain one β3 subunit harbouring the E77K substitution, whereas ‘double-variant’ receptors contain E77K substitutions in both β3 subunits. Changes in GABA sensitivity were quantified by calculating the logarithmic shift in EC_50_ values (ΔlogEC_50_) for each experimental day. Cells expressing the β3^E77K^ mutations exhibited a 3- to 5-fold increase in GABA sensitivity, as reflected by ΔlogEC_50_ values of 0.54 ± 0.06 for single-variant and 0.69 ± 0.082 for double-variant α1β3γ2 receptors, compared with WT (Fig. 1B and Supplementary Table 4). Further analysis of macroscopic currents revealed changes in current decay kinetics (Fig. 1B, inset, and Supplementary Table 4). The fastest decay rate was observed in the double-mutant receptor, with a decay constant (*k*) of 0.023 ± 0.005 s^−1^, compared with 0.014 ± 0.002 s^−1^ for the WT receptor. The single-mutant receptor exhibited an intermediate *k* value of 0.018 ± 0.005 s^−1^. These findings are consistent with previous reports and indicate that β3^E77K^-containing α1β3γ2 GABA_A_ receptors display a GOF molecular phenotype with modest but measurable acceleration of decay kinetics.^2,11,17^

### Embryonic mortality, reduced body weight and normal brain size in β3^E77K^-6J mice

Heterozygous β3^E77K^-6J mice were not born at Mendelian ratios. Genotyping at embryonic day 15 (E15) revealed that the β3^E77K^-6J variant was present in 25% of pups, indicating embryonic lethality or potentially prefertilization issues (Fig. 1C). Likewise, genotyping at P7 showed that 21% of pups carried the variant (Fig. 1C). Surviving β3^E77K^-6J mice were significantly smaller than WT littermates from P7 to P21 (Fig. 1D and E). Brain weight and gross brain morphology showed no differences between β3^E77K^-6J and WT mice (Fig. 1F and G). Other morphological markers of neuronal excitability, including staining for mossy fibre sprouting (ZnT3),^23,24^ neurodegeneration (Fluorojade),^25^ myelin basic protein^26^ and Iba-1,^27^ were not different between β3^E77K^-6J and WT mice (Fig. 1H and Supplementary Fig. 2).

### β3^E77K^-6J mice exhibit spike-wave-like activity and cortical hyperexcitability

β3^E77K^-6J mice showed an increased propensity for premature death without hindlimb extension, which occurred over ages ranging from P50 to P150 (Fig. 2A). Despite extensive video monitoring, overt spontaneous seizures were not observed. To ascertain whether β3^E77K^-6J mice were more susceptible to seizures, we used a standard proconvulsant test based on the GABA_A_ receptor antagonist, PTZ.^28-30^ We measured the time to two well-defined seizure end points: the first tonic–clonic seizure and hindlimb extension following the subcutaneous injection of PTZ (100 mg kg^−1^).^28^ Both the time to first tonic–clonic seizure and hindlimb extension were significantly shorter in β3^E77K^-6J mice (Fig. 2B).

**Figure 2:**
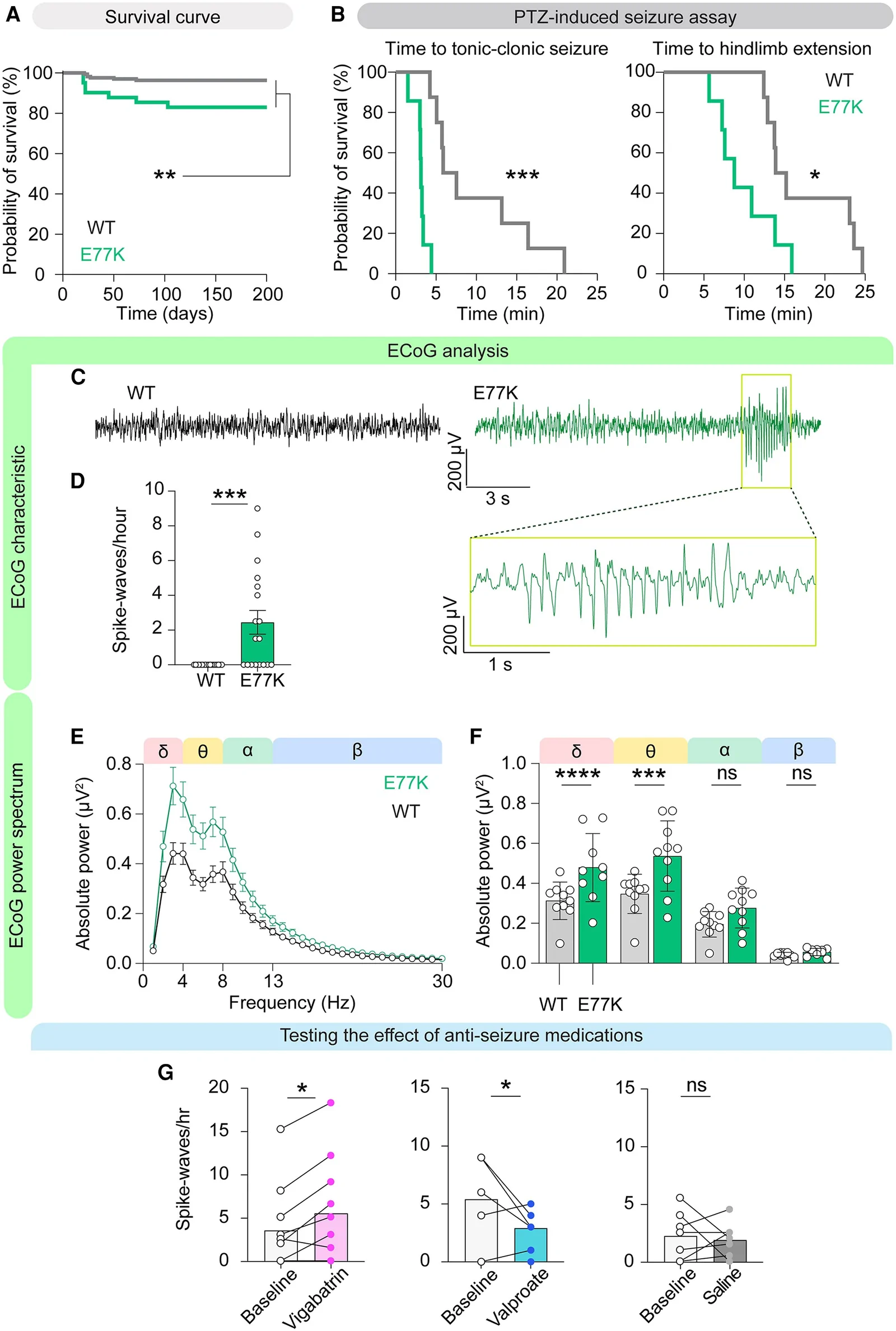
**β3^E77K^-6J mice exhibit reduced survival probability, increased epileptiform activity and elevated cortical spectral power, and they reproduce the response of patients to anti-seizure medications.** (**A**) β3^E77K^ mice showed significantly reduced survival compared with wild-type (WT) littermates, *n* = 165 WT and 41 E77K, **P = 0.0012, Mantel–Cox log-rank test. (**B**) In the subcutaneous pentylenetetrazole (PTZ) assay, β3^E77K^ mice had a significantly shorter time to first tonic–clonic seizure (*n* = 8 per group, **P = 0.0025) and hindlimb extension (*P = 0.0213), Mantel–Cox log-rank test. (**C**) Representative electrocorticography (ECoG) traces from a WT mouse (*left*, black) and a β3^E77K^ mouse (*right*, green) showing a spike-wave-like discharge. The boxed region highlights an individual spike-wave-like complex, which is expanded in the lower trace. (**D**) Quantification of spike-wave-like discharges (SWDs) per hour revealed a significant increase in β3^E77K^ mice compared with WT (*n* = 18 per group, ***P = 0.0003, Mann–Whitney U-test). (**E**) Representative ECoG power spectrum recorded during the light phase, illustrating elevated power across all frequencies in β3^E77K^ mice. (**F**) Mean absolute ECoG spectral power during the light phase was significantly elevated in β3^E77K^-6J mice compared with WT. Two-way repeated-measures ANOVA revealed a significant main effect of genotype [*F*(3,36) = 33.21, ****P < 0.0001], frequency band [*F*(1,36) = 30.94, ****P < 0.0001], and a Genotype × Band interaction [*F*(3,36) = 4.45, **P = 0.0093]. Holm–Šidák *post hoc* tests confirmed significantly increased delta (adjusted ****P < 0.0001) and theta power (adjusted ****P = 0.0006) in β3^E77K^ mice, with no significant difference in alpha or beta bands (adjusted *P* = 0.1654 and *P* = 0.7408, respectively). *n* = 10 per group. (**G**) Drug modulation of SWD frequency in β3^E77K^-6J mice. *Left*: Vigabatrin (300 mg kg^−1^, intraperitoneal) significantly increased SWD frequency compared with baseline recordings (*n* = 10, *P = 0.0312, Wilcoxon test), consistent with exacerbation of epileptiform activity. *Middle*: Valproate (200 mg kg^−1^, intraperitoneal) significantly reduced SWD frequency compared with baseline (*n* = 8, *P = 0.0410, Student’s paired *t*-test). *Right*: Vehicle control (saline, intraperitoneal) did not alter SWD frequency compared with baseline (*n* = 7, *P* = 0.7143, Student’s paired *t*-test). Data are presented as the mean ± standard error of the mean for power spectrum and mean ± standard deviation for all other graphs.

To determine whether GOF β3^E77K^-6J mice had electrographic signs of brain excitability, we performed electrocorticography (ECoG) recordings. Baseline ECoG traces from β3^E77K^-6J mice revealed distinct spontaneous epileptiform activity, including spike-wave-like discharge (SWD) events not observed in WT mice (Fig. 2C and D). SWD morphological characteristics comprised three or more spike–wave cycles at 7–10 Hz and event duration of 0.3–2.0 s, consistent with other animal models of absence seizures.^31^ These spontaneous SWD events were detected in approximately half of the β3^E77K^-6J mice during the 4 h baseline recording period. Given the brief duration of the events, we were unable to measure consistent stereotypical behaviours with simultaneous video monitoring. Accordingly, these events were classified as spike-wave-like discharges rather than absence seizures.

Analysis of ECoG spectral power revealed a significant increase in absolute power in β3^E77K^-6J mice, driven primarily by elevations in the delta (1–4 Hz) and theta (4–8 Hz) frequency bands (Fig. 2E and F and Supplementary Fig. 3). In contrast, power in the alpha (8–12 Hz) and beta (12–30 Hz) bands did not differ significantly between genotypes, despite showing a similar directional trend. Enhanced slow-frequency activity has been linked to increased seizure susceptibility in both experimental models and clinical epilepsy.^32^ Power spectra were calculated from fixed 30 min recording epochs selected at defined circadian time points during the light and dark phases, ensuring consistent sampling across genotypes.

Interestingly, vigabatrin (300 mg kg^−1^, intraperitoneally), which enhances GABAergic tone by inhibiting GABA transaminase and blocking GABA breakdown,^33,34^ increased SWD count in 6 of 10 mice (Fig. 2G) in line with adverse events observed in *GABRB3* GOF patients by this drug,^11^ while valproate (200 mg kg^−1^, intraperitoneally) reduced the number of events (Fig. 2G).

### β3^E77K^-6J mice have reduced locomotor activity and weakened grip strength

A series of behavioural tests were performed on β3^E77K^-6J mice and their WT littermates (Fig. 3 and Supplementary Table 5). Interestingly, in contrast to the hyperactivity observed in the LOF β3^D120N^ and β3^N110D^ mouse models,^14,15^ β3^E77K^-6J mice exhibited marked hypoactivity. They showed a significant reduction in vertical jump counts at both young (P21–P34) and adult (P45–P60) ages, and there was also a significant reduction in distance travelled at the older age (Fig. 3A and B). This hypoactivity was not attributable to a loss of gross motor control, because β3^E77K^-6J mice performed similarly to their WT littermates in the rotarod and ledged beam tests (Fig. 3C and D). The β3^E77K^-6J mice did, however, have a reduced grip-strength phenotype, indicating a mild motor weakness distinct from general locomotor activity (Fig. 3E). Four tests were performed to probe anxiety-like behaviours. β3^E77K^-6J mice travelled less in the centre zone of the locomotor cell, spent more time in the light zone during the light–dark test and spent equal time in the open arms of the elevated plus maze (Fig. 3F–H). These observations are inconsistent with any clear anxiety profile but do suggest distinct behavioural phenotypes in the β3^E77K^-6J mice. Next, the three-chamber test was used to probe social interaction, with β3^E77K^-6J mice spending similar time with a stranger mouse to their WT littermates, suggesting no deficit (Fig. 3I). Finally, no gross memory deficits were observed in the β3^E77K^-6J mouse model when probed using the Barnes maze and the Y-maze tests (Supplementary Fig. 4).

**Figure 3:**
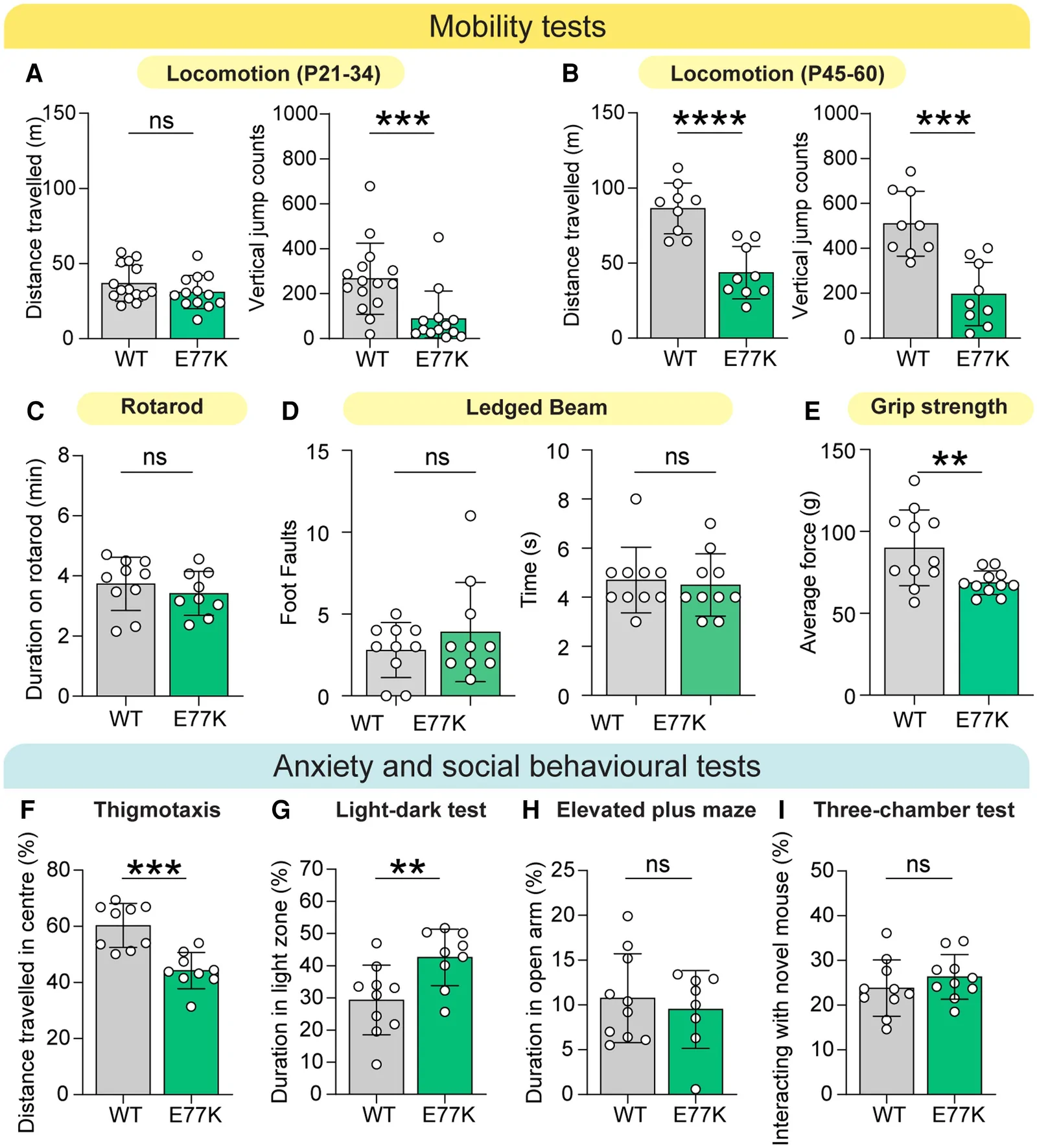
**β3^E77K^-6J mice display alterations in behaviour compared with wild-type littermates.** (**A**) In juvenile mice postnatal Days 21–34, β3^E77K^ mice showed no significant difference in total distance travelled compared with wild-type (WT) littermates but exhibited a significant reduction in vertical jump counts (*n* = 15 WT, *n* = 13 β3^E77K^; *P* = 0.2539 and ***P = 0.0008, respectively, Mann–Whitney U-tests). (**B**) In older mice (P45–P60), both total distance travelled and vertical jump counts were significantly reduced in β3^E77K^ mice (*n* = 9 WT, *n* = 9 β3^E77K^; ****P < 0.0001, ***P = 0.0003, Student’s unpaired *t*-tests). (**C**) Time spent on the rotarod was not significantly different (*n* = 10 WT, *n* = 10 β3^E77K^; *P* = 0.4077). (**D**) In the ledged beam test, there were no significant differences between genotypes in the number of foot faults (*n* = 10 per group; *P* = 0.8631, Mann–Whitney U-test) or the time taken to traverse the beam (*P* = 0.6939). (**E**) Grip strength was significantly reduced in β3^E77K^ mice compared with WT littermates (*n* = 11 per group; **P = 0.0088, Student’s unpaired *t*-test). (**F**) β3^E77K^ mice travelled significantly less within the centre zone of the locomotor activity chamber, (*n* = 9 WT, *n* = 9 β3^E77K^; ***P = 0.0002, Student’s unpaired *t*-test). (**G**) In the light–dark box assay, β3^E77K^ mice spent more time in the light zone (**P = 0.0097, Student’s unpaired *t*-test). (**H**) No differences were observed between genotypes in the elevated plus maze (*n* = 10 WT, *n* = 8 β3^E77K^; *P* = 0.5835) or (**I**) in the three-chamber social interaction test (*n* = 10 WT, *n* = 10 β3^E77K^; *P* = 0.3421). Data are presented as the mean ± standard deviation.

Taken together, β3^E77K^-6J mice replicate some key features of GOF *GABRB3* disease, including generalized SWDs, worsening of the SWD phenotype with vigabatrin, increased proconvulsant seizure susceptibility, hypoactivity and early mortality risk. We next explored synaptic level changes that underlie phenotypic alterations associated with GOF *GABRB3* variants.

### β3^E77K^-6J CA1 neurons display increased GABA_A_ receptor-mediated synaptic current amplitudes

To determine whether the changes in the biophysical properties of β3^E77K^-containing GABA_A_ receptors translate to the synaptic level, we performed whole-cell electrophysiological recordings within the CA1 hippocampal region of β3^E77K^-6J and WT mice (Fig. 4 and Supplementary Table 3). mIPSCs were recorded at −70 mV in the presence of blockers of excitatory synaptic transmission and sodium channels using a high chloride-based internal solution. A sliding template algorithm was used to identify mIPSCs.

**Figure 4:**
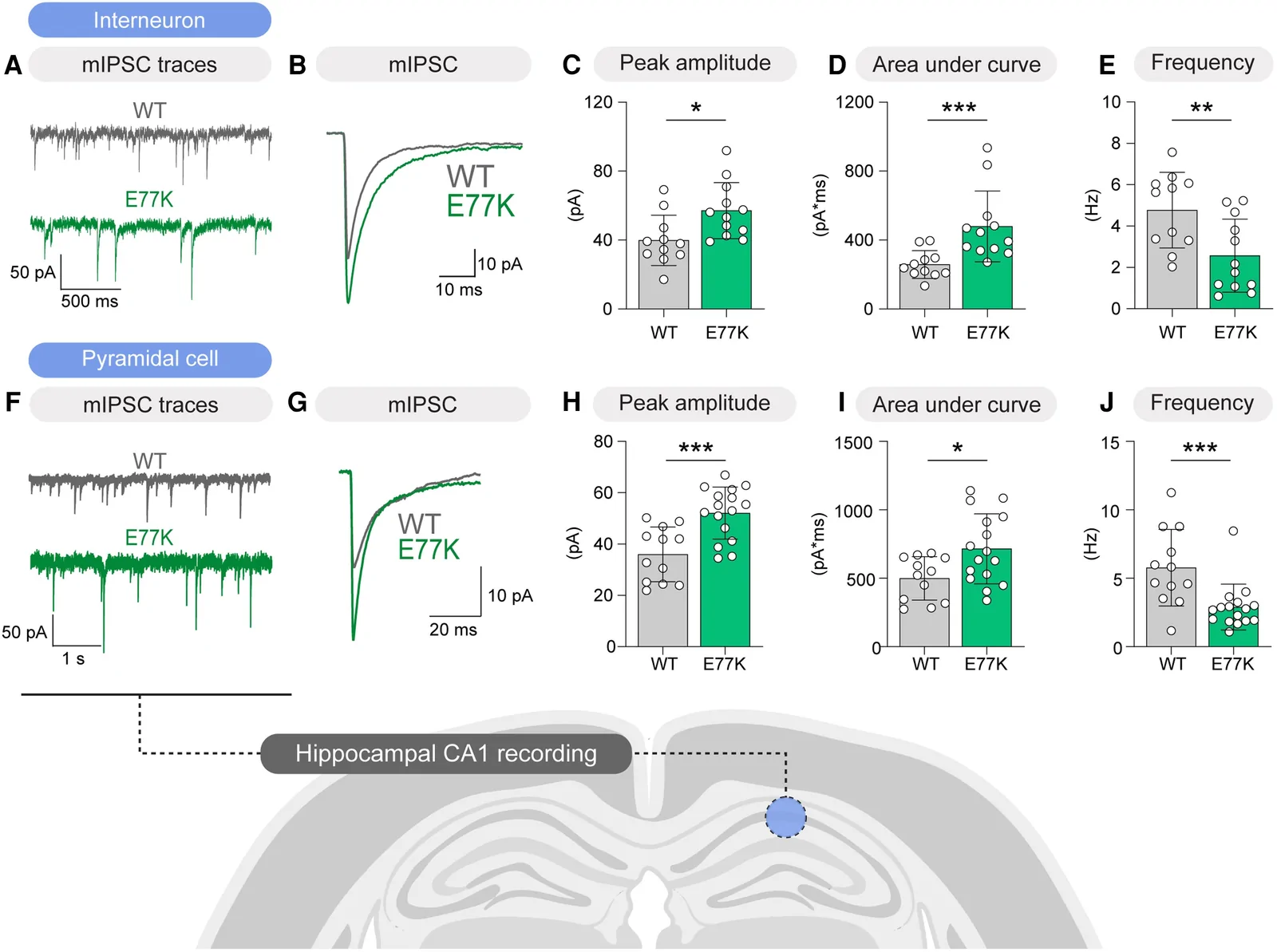
**β3^E77K^-6J mice have enhanced inhibitory synaptic transmission in CA1 hippocampal neurons.** (**A**) Miniature inhibitory postsynaptic current (mIPSC) traces from both wild-type (WT; grey) and β3^E77K^ (green) mice in CA1 hippocampal interneurons. (**B**) Overlaid averaged mIPSC traces. (**C**)  β3^E77K^ mice (*n* = 12) have significant increases in averaged mIPSC peak amplitude compared with the WT mice (*n* = 11; *P = 0.0142, Student’s unpaired *t*-test). (**D**) The area under the curve of mIPSCs was also significantly higher in β3^E77K^ mice compared with the WT mice (***P = 0.0005, Mann–Whitney U-test). (**E**) Miniature IPSC frequency was lower in the β3^E77K^ mice (**P = 0.0080, Student’s unpaired *t*-test). (**F**) mIPSC traces from both WT (grey) and β3^E77K^ (green) mice in CA1 pyramidal neurons. (**G**) Overlaid averaged mIPSC traces. (**H**–**J**) Similar to the hippocampal interneurons, both mIPSC peak amplitudes and area under curve were significantly higher in the β3^E77K^ mice (*n* = 16) compared with the WT mice (*n* = 12; ***P = 0.0004 and *P = 0.0162, respectively, Student’s unpaired *t*-test). Miniature IPSC frequency was also significantly lower in β3^E77K^ mice compared with WT (***P = 0.0008, Mann–Whitney U-test). Data are presented as the mean ± standard deviation.

The average amplitude and area under the curve of mIPSCs were significantly larger in CA1 stratum oriens interneurons from β3^E77K^-6J mice, consistent with a GOF phenotype (43% increase in amplitude and 85% increase in area under the curve; Fig. 4A–D). This was accompanied by a lower mIPSC frequency, potentially attributable to adaptive processes (46% decrease; Fig. 4E). The synaptic profile of CA1 pyramidal neurons was similar to that observed in inhibitory neurons, including increased mIPSC amplitude and area under the curve (45% increase in amplitude and 43% increase in area under the curve; Fig. 4F–I) and reduced frequency (50% decrease; Fig. 4J). These data are consistent with the biophysical GOF characteristics observed for β3^E77K^ mutated channels in the oocyte expression system (Fig. 1B).^2,11,17^

### Differential GABA_A_ receptor-mediated synaptic currents in layer 2/3 β3^E77K^-6J neurons

Next, we investigated synaptic function within layer 2/3 of the somatosensory cortex. Layer 2/3 inhibitory neurons were identified based on laminar location, basket-cell morphology and low membrane capacitance, with interneuron identity further supported by *post hoc* PV immunohistochemistry in a subset of recordings (see the ‘Materials and Methods’ section, Fig. 5A, Supplementary Fig. 5 and Supplementary Table 3). The average amplitude and area under the curve of captured mIPSC events were larger in β3^E77K^-6J than WT inhibitory neurons (68% increase in amplitude, 59% increase in area under the curve; Fig. 5B–G). No significant differences were observed in mIPSC frequency (Fig. 5H). There was no difference in the action potential firing frequency–input relationship for fast-spiking interneurons (Fig. 5I–M).

**Figure 5:**
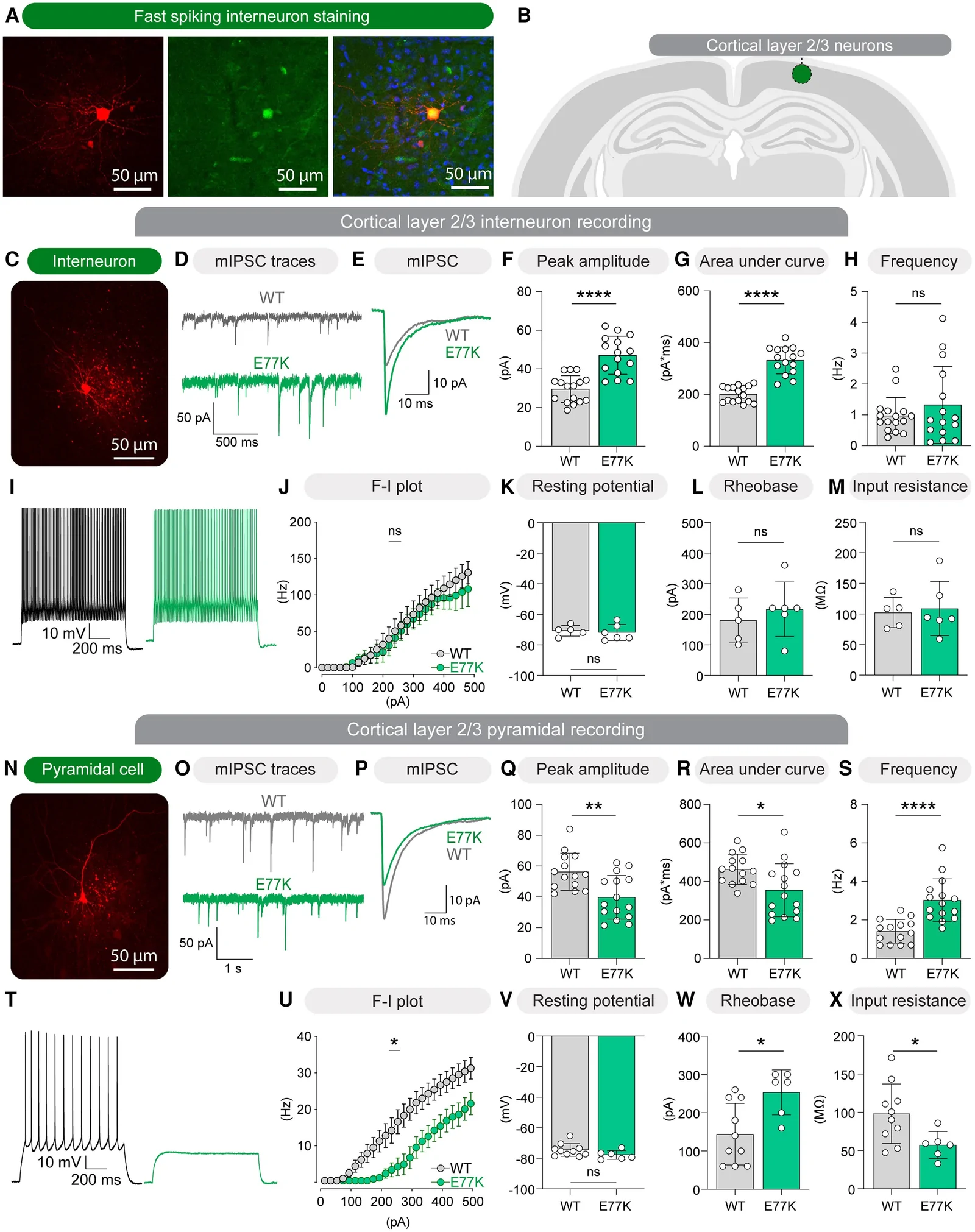
**β3^E77K^-6J mice show enhanced inhibitory synaptic transmission in cortical interneurons and altered intrinsic properties in pyramidal neurons**. (**A**) Representative immunofluorescent images of a fast-spiking interneuron showing a biocytin-filled cell with streptavidin staining (red; *left*), parvalbumin (PV) staining (green; *middle*), and merged, overlaid with DAPI (blue; *right*). (**B**) Schematic illustration of recording site within layer 2/3 of the somatosensory cortex. (**C**) Example image of a patched fast-spiking interneuron. (**D**) Representative miniature inhibitory postsynaptic current (mIPSC) traces recorded from layer 2/3 interneurons in wild-type (WT; grey) and β3^E77K^ (green) mice. (**E**) Averaged, overlaid mIPSC traces from WT and β3^E77K^ interneurons. (**F**–**H**) Summary of mIPSC parameters recorded from interneurons, showing significantly increased peak amplitude (****P < 0.0001, Student’s unpaired *t*-test) and area under the curve (****P < 0.0001, Mann–Whitney U-test) in β3^E77K^ mice compared with WT, with no difference in event frequency (*P* = 0.32, Mann–Whitney U-test). *n* = 16 WT, *n* = 15 E77K for **F**–**H**. (**I**) Example firing trace from an interneuron at 280 pA current injection. (**J**–**M**) Interneuron intrinsic membrane properties, including frequency–current (*F–I*) relationship (*P =* 0.6561, mixed model analysis), resting membrane potential (*P =* 0.6918), rheobase (*P =* 0.4812, Student’s unpaired *t*-tests) and input resistance (*P =* 0.7839), were not significantly different between genotypes. *n* = 5 WT and *n* = 6 E77K for **J**–**M**. (**N**) Representative image of a layer 2/3 pyramidal neuron from a β3^E77K^ mouse. (**O**) Representative mIPSC traces recorded from pyramidal neurons in WT (grey) and β3^E77K^ (green) mice. (**P**) Averaged, overlaid mIPSC traces from WT and β3^E77K^ pyramidal neurons. (**Q**–**S**) β3^E77K^ pyramidal neurons displayed significantly smaller mIPSC peak amplitude (**P = 0.0023) and area under the curve (*P = 0.0154), but significantly higher mIPSC frequency (****P < 0.0001, Student’s unpaired *t*-tests). (**T**) Example firing traces from a pyramidal neuron at 140 pA current injection. (**U**–**X**) β3^E77K^ cells showed a right-shifted *F–I* relationship (*P = 0.0214, mixed model analysis), unchanged resting membrane potential (*P* = 0.1121), increased rheobase (*P = 0.0121, Student’s unpaired *t*-tests) and reduced input resistance (*P = 0.0297). *n* = 10 WT and *n* = 6 E77K for **U**–**X**.

In contrast, mIPSC amplitude and area under the curve were smaller from β3^E77K^-6J than WT pyramidal neurons (29% decrease in amplitude, 23% decrease in area under the curve; Fig. 5N–R). This was, however, accompanied by an increase in mIPSC frequency in β3^E77K^-6J cells (114% increase; Fig. 5S). Hence, layer 2/3 inhibitory neurons and pyramidal neurons exhibit differential changes of inhibitory synaptic current amplitude in β3^E77K^-6J mice. Tonic GABA_A_ receptor-mediated currents were also measured in layer 2/3 pyramidal neurons and were not significantly different between genotypes (Supplementary Fig. 6). The frequency–input action potential firing relationship for pyramidal neurons was right shifted (Fig. 5T–X), with a significant increase in current injection amplitude required to reach rheobase in β3^E77K^-6J cells (Fig. 5W), probably attributable to a reduction in input resistance (Fig. 5X).

To assess whether excitatory synaptic transmission was altered, spontaneous excitatory postsynaptic currents (EPSCs) were recorded from layer 2/3 interneurons and pyramidal neurons. No significant differences were observed in spontaneous EPSC amplitude, frequency or kinetics across genotypes (Supplementary Fig. 7), indicating that glutamatergic transmission remains largely unaltered within this brain region in the β3^E77K^-6J mice.

Finally, to investigate whether differences in GABA_A_ receptor subunit expression might underlie the distinct effects on inhibitory synaptic transmission between interneuron and pyramidal neuron synapses, we analysed published single-cell RNA-sequencing data obtained from mouse somatosensory cortex.^21^ Expression levels of all GABA_A_ receptor subunit mRNA were similar in PV^+^ interneurons and *Cux2*-identified pyramidal neurons (Supplementary Fig. 8 and Supplementary Table 6). This suggests that differential mRNA expression for distinct GABA_A_ receptor subunits is unlikely to account for the synapse-specific impact of the β3^E77K^-6J variant.

### Collapsing feed-forward inhibition underlies cortical hyperexcitability in GOF *GABRB3* disease

Next, we explored the dynamic consequences of disrupted inhibition within the somatosensory cortex. Pyramidal neuron excitability was probed by recording the membrane potential in response to a cortical excitability quantification protocol.^35^ This protocol involves recording the response of a pyramidal neuron to a single shock applied to layer 4 excitatory axons both before and 100 ms after a conditioning stimulatus train applied to a different set of layer 4 excitatory axons (Fig. 6). Stimulation of layer 4 excitatory input (using an AP threshold stimulus) activates the pyramidal neuron through direct synaptic input but also recruits multiple interneurons, which provide feed-forward inhibitory input to the pyramidal neuron.^35^ In WT cells, the inhibition induced by the conditioning stimulus persists for >100 ms following the stimulus train, leading to a substantial reduction in firing probability.^36^ This effect was evident from the significant decrease in action potentials generated after the conditioning train (50% decrease; Fig. 6A and B). In contrast, β3^E77K^-6J cells exhibit a higher number of action potentials following the conditioning train, indicative of reduced inhibitory control in layer 2/3 pyramidal cells and heightened cortical excitability (29% increase; Fig. 6C and D).

**Figure 6:**
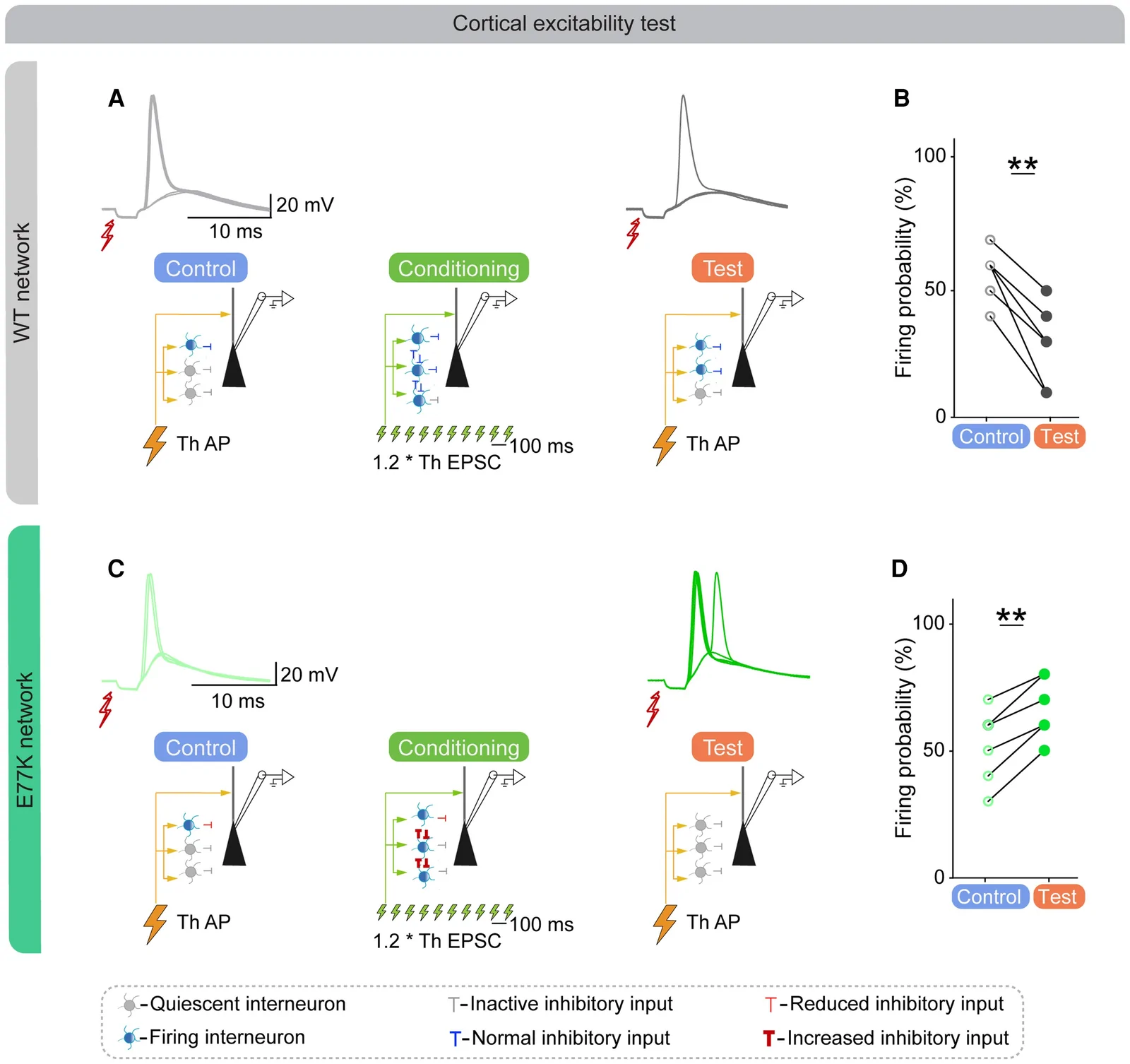
**Disinhibition of interneurons underlies the increased firing probability in β3^E77K^-6J network.** (**A**) *Top*: Example action potential (AP) elicited by stimulation of L4 input in a wild-type (WT) mouse in control conditions (light grey). Three of five trials resulted in AP firing. *Bottom*: Network model in control conditions, with direct excitatory input and weak bi-synaptic inhibitory input. *Middle*: Schematic diagram of the network model during conditioning, showing multiple interneurons being recruited, resulting in stronger feed-forward inhibition. *Top right*: Example AP elicited in the same neuron in test conditions (dark grey). One of five trials resulted in AP firing. *Bottom right*: Network model in test condition, with direct excitatory input and stronger feed-forward inhibitory input in WT network. (**B**) Statistical analysis showing the reduced firing probability in the WT network (*n* = 12 trials from six pyramidal cells, **P = 0.0019, Student’s paired *t*-test). (**C**) *Top*: Example APs elicited in β3^E77K^ mouse in control conditions (light green). Two of five trials resulted in AP firing. *Bottom*: Network model in control conditions of β3^E77K^ network showing reduced inhibitory input. *Middle*: Schematic diagram of the network model during conditioning, showing fewer interneurons being recruited owing to strong disinhibition in feed-forward network, resulting in weaker feed-forward inhibitory input. *Top right*: Example APs elicited in the same neuron in test conditions (dark green). Four of five trials resulted in AP firing. *Bottom right*: Network model in control conditions of β3^E77K^ network, showing reduced inhibitory input. (**D**) AP firing frequency was increased in the β3^E77K^ mouse (*n* = 12 trials from six cells, **P = 0.0011, Student’s paired *t*-test). ESPC = excitatory postsynaptic current; Th = stimulation threshold.

### Behavioural phenotypes and impaired cortical feed-forward inhibition in β3^E77K^-6N mice

The β3^E77K^ line was initially generated and characterized on a C57Bl/6J background, where behavioural and electrographic phenotypes were robust. However, this background was also associated with pronounced embryonic lethality and early mortality. Given established phenotypic differences between C57Bl/6 substrains,^37^ we therefore established a parallel β3^E77K^ line on a C57Bl/6N background to assess strain dependence and to determine whether the core disease phenotypes were preserved in conditions of improved survival. WT C57Bl/6N mice were crossed with β3^E77K^ mice, and β3^E77K^-6N was created. Notably, in β3^E77K^-6N mice, embryonic lethality was less evident in the F1 generation, suggesting a milder impact of the GOF variant on the β3^E77K^-6N background (Supplementary Fig. 9). To explore the impact of background strain on β3^E77K^ behavioural phenotypes further, locomotor activity was assessed in β3^E77K^-6N adult mice (P40). In comparison to WT controls, β3^E77K^-6N mice had reduced vertical jump counts but travelled a similar distance (Supplementary Fig. 9 and Supplementary Table 7), indicating a milder locomotor impairment relative to β3^E77K^-6J mice (Fig. 3B). Interestingly, β3^E77K^-6N mice displayed a pronounced hindlimb clasping phenotype, which was absent in β3^E77K^-6J mice (Supplementary Fig. 10 and Supplementary Table 7). Importantly, β3^E77K^-6N mice displayed recurrent SWDs and increased ECoG power, closely mirroring the cortical hyperexcitability seen in β3^E77K^-6J mice (Supplementary Figs 9C–F and 3, respectively). Overall, β3^E77K^-6N mice recapitulated the core behavioural, electrographic and synaptic phenotypes observed in β3^E77K^-6J mice, albeit with reduced severity, indicating that genetic background modulates phenotypic expression without altering the underlying disease mechanism.

Single-cell recordings revealed that at 1.2 × threshold stimulation of L4 axons, the amplitude of disynaptic IPSCs onto layer 2/3 pyramidal neurons was smaller in β3^E77K^-6N neurons (36% decrease; Fig. 7). This is consistent with the smaller mIPSCs recorded in the β3^E77K^-6J mice (Fig. 5N–R) and suggests that GABA_A_ receptor-mediated inhibition was significantly compromised in layer 2/3 pyramidal neurons, both at the synaptic scale and through deficits at the local micro-network scale. EPSC amplitudes were unchanged, resulting in an increased excitatory-to-inhibitory ratio (Fig. 7). These data confirm a selective reduction in inhibitory drive without a compensatory change in excitation at the synaptic level.

**Figure 7:**
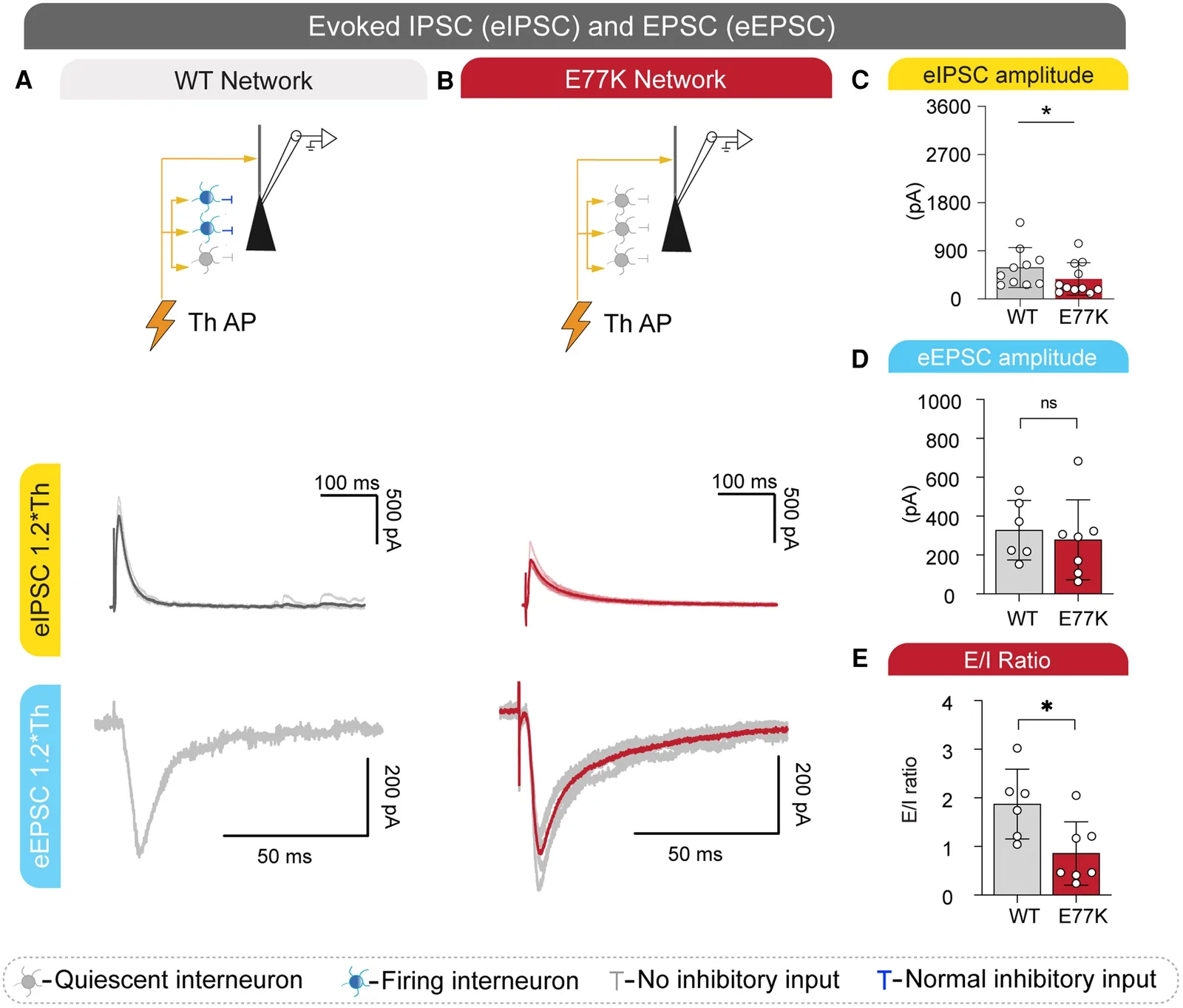
**Reduced layer 4-evoked inhibitory drive and increased excitatory-to-inhibitory ratio in β3^E77K^-6N mice.** (**A**) *Top*: Schematic diagram of the network model in wild-type (WT) mice, showing direct excitatory input and normal inhibitory input to layer 2/3 (L2/3) pyramidal neurons. *Middle*: Representative voltage-clamp recordings (holding potential = 0 mV) show disynaptic evoked inhibitory postsynaptic currents (eIPSCs) in response to layer 4 (L4) stimulation at 1.2 × threshold (Th) in a L2/3 pyramidal neuron. Five superimposed sweeps are shown, with the average trace in bold. *Bottom*: Representative evoked excitatory postsynaptic currents (eEPSCs) recorded at −70 mV from the same cells. (**B**) Same as in **A**, recorded from a β3^E77K^-6N mouse. (**C**) Summary of eIPSC amplitudes evoked by L4 stimulation at 1.2 × Th in WT (*n* = 10) and β3^E77K^ mice (*n* = 11; *P = 0.04, Mann–Whitney U-test). (**D**) Summary of eEPSC amplitudes (WT: 327 ± 62 pA, *n* = 7; E77K: 278 ± 78 pA, *n* = 6; *P* = 0.27, Mann–Whitney U-test), showing no difference between genotypes. (**E**) Excitatory-to-inhibitory (E/I) ratio calculated from the paired eEPSC and eIPSC amplitudes (WT: 0.86 ± 0.25; E77K: 1.87 ± 0.29; *P = 0.01, Mann–Whitney U-test). Data are presented as the mean ± standard deviation. Th = stimulation threshold.

Importantly, analysis of activity-dependent feed-forward inhibition in β3^E77K^-6N mice revealed a deficit identical to that seen in β3^E77K^-6J mice (Supplementary Fig. 9). In WT neurons, a conditioning pulse increased inhibition, reducing the probability of AP firing in response to a subsequent test pulse (30% decrease; Supplementary Fig. 9). In stark contrast, the same conditioning pulse increased the probability of AP firing in β3^E77K^-6N mice (79% increase; Supplementary Fig. 9), recapitulating this key cellular phenotype across different mouse background strains.

### Simulations support impaired feed-forward inhibition

We hypothesized that enhanced inhibitory-to-inhibitory synaptic interactions can disrupt cortical layer 2/3 feed-forward inhibition. To test this hypothesis, we created a simple feed-forward network model containing a basket cell population, a pyramidal cell and physiologically realistic synaptic latencies,^38^ and we applied a similar conditioning and test stimulus to the experimental protocol (Fig. 8). The presence of a conditioning stimulus suppressed pyramidal cell spiking through feed-forward inhibition in the WT network (Fig. 8A). However, enhancing the amplitude of inhibitory-to-inhibitory GABA_A_ receptor synaptic conductance to model the impact of β3^E77K^ increased pyramidal cell spiking and was associated with reduced inhibitory synaptic conductance onto the pyramidal cell consistent with a disinhibitory influence (Fig. 8B and C).

**Figure 8:**
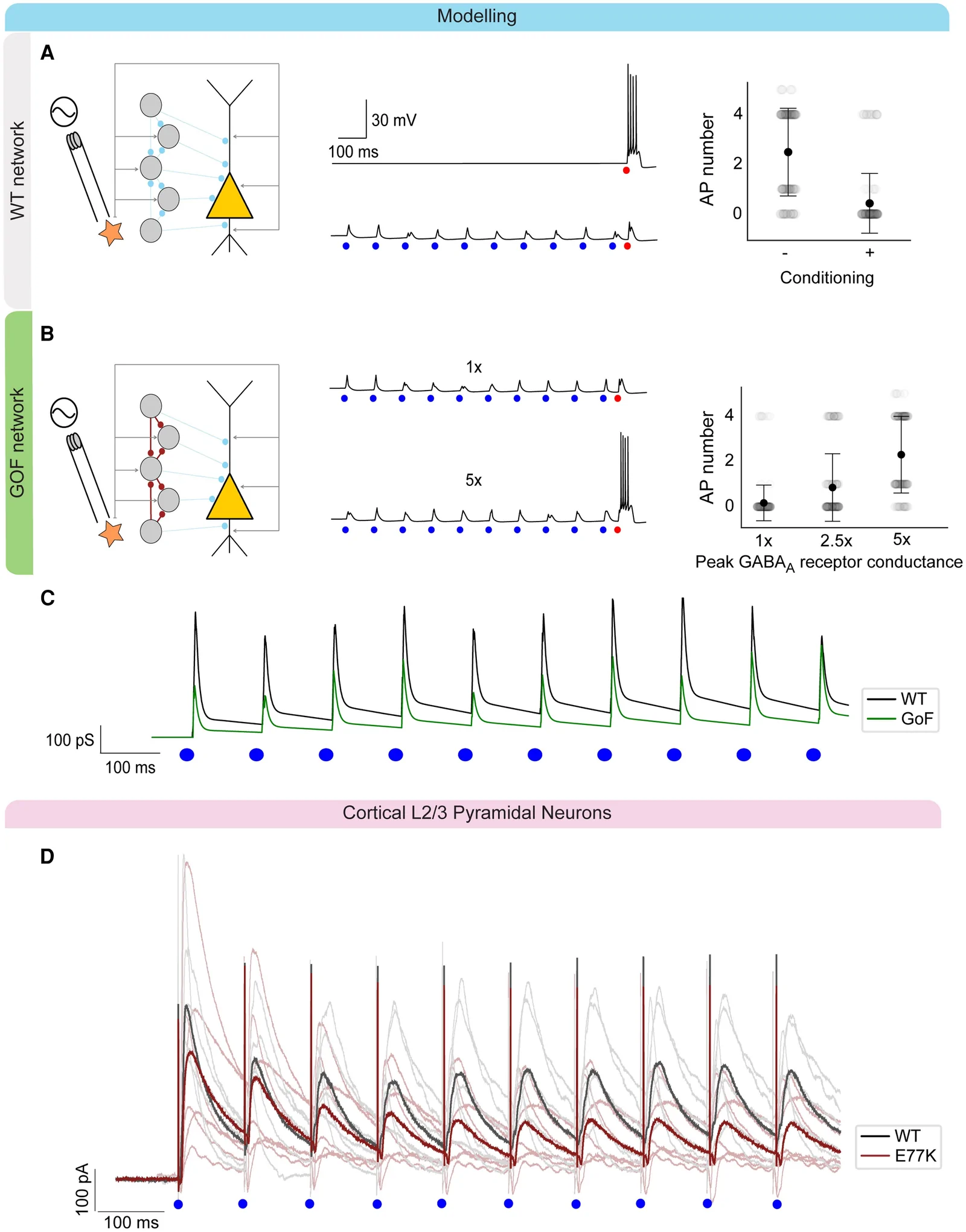
**Network model of feed-forward disinhibition and inhibitory postsynaptic current during conditioning in β3^E77K^-6N mice.** (**A**) Schematic diagram of wild-type (WT) network (*left*) and representative time–voltage traces from a pyramidal cell (PC) without (*middle*, *top*) and with (*middle*, *bottom*) conditioning stimuli. Application of the conditioning stimuli suppressed PC action potential (AP) spiking, consistent with feed-forward inhibition mediated by the basket cell (BC) population (*right*). (**B**) Schematic diagram of gain-of-function (GOF) network and representative time–voltage traces from a PC comparing 1×, 2.5× and 5× peak GABA_A_ receptor conductance of BC-to-BC synapses. Enhancing BC-to-BC synaptic conductance increased PC firing, consistent with feed-forward disinhibition (*right*). (**C**) A disinhibitory effect is confirmed when considering the mean PC GABA_A_ receptor conductance during conditioning stimuli for WT and GOF GABA_A_ receptors. Here, mean inhibitory conductance is suppressed in the GOF model compared with WT. (**D**) Inhibitory postsynaptic current (IPSC) recordings during the conditioning stimulus in β3^E77K^-6N cortical L2/3 pyramidal neurons at 0 mV holding potential (dark grey, average of six WT traces; dark red, average of six E77K traces). Similar to the model, lower inhibitory currents are observed in the E77K recordings.

To corroborate the simulation predictions, we assessed the impact of the *Gabrb3* E77K variant on IPSC magnitude during the conditioning stimulus that we recorded from β3^E77K^-6N L2/3 pyramidal neurons at a holding potential of 0 mV (Fig. 8D). Impaired feed-forward inhibition was evident in β3^E77K^-6N L2/3 pyramidal neurons, matching the predictions made from computer simulation modelling (Fig. 8C). These findings strongly support the premise that enhancing GABA_A_ receptor function at PV^+^-to-PV^+^ interneuron synapses is sufficient to compromise activity-dependent feed-forward inhibition and provides a plausible mechanistic link between the GOF *GABRB3* variant and the emergence of cortical hyperexcitability.

## Discussion

The role of GABA_A_ receptor variants in causing epilepsy through a GOF mechanism has only recently been established, and it remains unclear how increased GABA_A_ receptor-mediated transmission leads to disease. We propose that this phenomenon arises from a collapse in feed-forward inhibition, caused by enhanced GABA_A_ receptor-mediated transmission between inhibitory interneurons in the cortex. This study presents evidence for this hypothesis using the β3^E77K^ mouse model of DEE, which harbours a GOF variant in *GABRB3*.

Mice engineered to carry GOF *GABRB3* variants that cause the most severe clinical phenotypes in humans proved to be lethal on the C57Bl/6J genetic background, precluding successful breeding. Given the number of reported LOF models,^14-16^ this finding underscores that GOF alterations tend to be more detrimental than LOF variants. Of the four different *GABRB3* variants attempted, only the β3^E77K^-6J mouse could be bred. Even then, these mice were not at Mendelian ratios embryonically, further suggesting severe disease phenotypes early in development. No gross alterations were observed in brain morphology or brain weight that could explain prenatal deaths. Interestingly, embryonic lethality was not as evident in β3^E77K^-6N mice, arguing that genetic background plays a role. Additionally, it is important to note that surviving mice (both β3^E77K^-6J and β3^E77K^-6N) might represent less severely affected individuals, introducing survival bias and masking early developmental effects.

Careful analysis of ECoG revealed epileptiform activity in β3^E77K^-6J and β3^E77K^-6N mice recapitulating aspects of the seizure phenotype of the patient. Spectral analysis of ECoG also revealed larger absolute power in both strains, with β3^E77K^-6J mice also being more susceptible to the proconvulsant PTZ, indicating increased cortical hyperexcitability. A similar broad-spectrum increase in power was observed for interictal ECoG in the LOF β3^D120N^ mouse model.^15^ This might reflect a common increase in cortical hyperexcitability in both GOF and LOF *GABRB3* disease, albeit through distinct mechanisms. The mild seizure phenotype in the β3^E77K^ mouse is not entirely unexpected, given that one individual with the *GABRB3* p.(Glu77Lys) variant was seizure free until the age of 16 years.^18^ Additionally, another individual was well controlled on a standard-of-care anti-seizure medication, valproate, which also reduced SWDs in β3^E77K^-6J mice.^11^ Interestingly, vigabatrin worsened the SWD phenotype in β3^E77K^-6J mice, broadly reflecting the adverse events observed with this drug in patients with *GABRB3* GOF variants.^11^ The mechanistic basis of the differential impact of valproate and vigabatrin on the SWD-like phenotype in β3^E77K^-6J mice is unknown. Although both vigabatrin and valproate increase brain GABA levels by inhibiting GABA transaminase, the mode of action of valproate is not fully understood and potentially includes multiple additional molecular targets.^39^ The basis of the divergence between the two anti-seizure medications might therefore reflect broader network-modulating actions of valproate, a hypothesis that requires testing.

The observed reduction in locomotion in the open-field test is a highly unusual finding for mice engineered to model monogenic DEE. Most knock-in mice based on pathogenic variants across various genes display a hyperactivity phenotype.^14,15,40-42^ This includes the LOF β3^D120N^ and β3^N110D^ mouse models.^14,15^ An exception is the LOF β3^N328D^ mouse model, which is reported to have a pronounced locomotion deficit in the elevated zero maze.^16^ However, the reported distance travelled is essentially zero, and other measured behaviours, including time in exploration (which requires movement), are not different between the β3^N328D^ and WT mice.^16^ This points to a technical issue. We therefore argue that hypoactivity in the β3^E77K^ mice is unique and in stark contrast to the hyperactivity observed in the LOF *GABRB3* mouse models.^14-16^ Additionally, β3^E77K^-6N mice showed a significantly increased clasping behaviour, indicating a disconnect between the premotor and motor cortices.^43^ This reflex parallels the hand-wringing behaviour displayed by patients with Rett syndrome, further aligning with a deficit in motor function.^43^ β3^E77K^-6J mice also exhibited reduced grip strength, consistent with a motor or muscle weakness in the absence of gross deficits in balance or coordination.^44^ Taken together, these behavioural features might relate to hypotonia and motor delay, which are commonly reported in individuals with GOF *GABRB3* receptor disease.^2,3^

The clear stratification of patients with DEE caused by LOF and GOF *GABRB3* variants points to distinct neuronal network-scale differences across the two groups.^2,3^ Our GOF β3^E77K^ mouse modelling confirms this, showing severe early developmental mortality and reduced locomotion, highlighting clear differences when compared with published LOF β3 mice.^14-16^ It is interesting to note that patients with LOF GABA_A_ receptor variants tend to feature febrile seizures as part of their clinical spectrum.^2,3^ Dravet syndrome is a DEE characterized by prolonged febrile seizures, life-long, treatment-resistant epilepsy and significant comorbidities, including intellectual and motor impairment, with reduced GABAergic neuronal activity also proposed as the underlying pathogenic mechanism.^45,46^ This might also relate to hyperactivity, which is a feature of both *SCN1A* Dravet and LOF *GABRB3* mouse models.^14,15,47,48^ These correlations suggest that diseases in which there is reduced GABA_A_ receptor-mediated ‘tone’ cause converging network-scale changes in excitability, resulting in fever-sensitive seizures and hyperactivity.

The impact of the β3^E77K^ mutated protein on inhibitory synaptic transmission was complex but included clear increases in GABA_A_ receptor-mediated current amplitudes at some synapses, consistent with observations in expression assays.^2,11,17^ Within cortical layer 2/3, synaptic GABA_A_ receptor-mediated current amplitudes were paradoxically smaller onto pyramidal neurons but larger at inhibitory-to-inhibitory neuron synapses. It is unclear why miniature and evoked IPSC amplitudes are smaller onto layer 2/3 pyramidal neurons, contrasting with the increased amplitudes seen at CA1 neuron and layer 2/3 interneuron synapses. Although this cannot explain the ‘use-dependent’ reduction in firing caused by the conditioning pulse, it does suggest that a baseline reduction in GABA_A_ receptor-mediated synaptic transmission to layer 2/3 pyramidal neurons might also contribute to increased excitability. Analysis of published single-cell WT mouse transcriptomics data^21^ does not indicate the make-up of GABA_A_ receptor subunits to be different between inhibitory and excitatory neurons in the somatosensory cortex. It should be noted that a proteomic approach is required to confirm this conclusion. There is a marked increase in mIPSC frequency at layer 2/3 pyramidal cells compared with layer 2/3 and CA1 interneuron synapses, where the mIPSC frequency is unchanged or reduced, respectively. This might lead to reduced amplitudes attributable to increased receptor desensitization, to which E77K-containing receptors are prone.^11,17^ Furthermore, given the highly plastic nature of GABA_A_ receptor expression and trafficking, it is also possible that adaptive changes differ at each recorded synapse. In addition, synapse-specific β3 expression influences IPSC kinetics,^49^ suggesting that altered subcellular localization of β3^E77K^ receptors could differentially affect synaptic and perisynaptic inhibition. Further adaptation can be seen in the firing properties of layer 2/3 pyramidal cells, where a significant right shift in rheobase is observed. Reduced intrinsic excitability is unlikely to drive input-dependent excitability and presumably reflects compensation within the layer 2/3 microcircuit in response to disrupted feed-forward inhibition.

Micro-network-scale analysis confirmed use-dependent reduction in feed-forward inhibition within layer 2/3. Engaging feed-forward inhibition through a conditioned stimulus of layer 4 axons reduces the probability of AP firing in layer 2/3 pyramidal neurons in a WT setting (Figs 6 and 7G and H).^35^ In contrast, the action potential firing probability increases in layer 2/3 pyramidal neurons of both the β3^E77K^-6J and β3^E77K^-6N mice in the same conditions (Figs 6 and 7I and J). Simulation of a feed-forward network model supports the hypothesis that increases in GABA_A_ receptor-mediated conductance at inhibitory-to-inhibitory synapses can impair feed-forward inhibition and enhance microcircuit excitability, consistent with the key experimental findings (Fig. 8). Importantly, evoked IPSCs recorded in β3^E77K^-6N layer 2/3 pyramidal neurons during the L4 conditioning pulses are reduced in amplitude, as predicted by our simulations, suggesting that enhanced synaptic strength between PV^+^ interneurons alone can explain the increase in activity-dependent firing compared with WT (Figs 6, 7G–J and 8A and B).

Although we show that reduced inhibition predisposes to excitability in the β3^E77K^ mouse cortex, the involvement of inhibitory-to-inhibitory synapses and the dynamic nature of this reduction exerts different physiological consequences. In the LOF context, loss of GABA_A_ receptor activity at inhibitory-to-inhibitory synapses is likely to increase as neuronal activity engages more feed-forward interneurons. In stark contrast, in layer 2/3 of the GOF β3^E77K^ mouse models, feed-forward inhibition is disrupted in a way that leads to increasing activity-dependent inhibitory network inhibition, with interneurons becoming less active as network activity increases, effectively ‘collapsing’ inhibitory control. This divergence in feed-forward inhibitory control might contribute to key differences in clinical phenotypes seen between the groups.

The involvement of inhibitory-to-inhibitory synaptic interactions also has interesting implications for network excitability that might contribute to phenotypic differences between LOF and GOF variants. LOF and GOF at inhibitory-to-inhibitory synapses lead to reduced and increased excitability in feed-forward inhibitory networks, respectively, whereas their impact is more complex within recurrent neural circuits. Here, LOF has been shown to predispose to network instability in the presence of strong excitatory-to-excitatory connections, and GOF to enhanced firing rates without necessarily causing instability.^50^ Therefore, it is possible that LOF and GOF variants that disrupt inhibitory-to-inhibitory synapses might differentially impact network excitability according to the prevailing region-specific neural circuit motif. Circuits within the prefrontal cortex, for example, are known to possess strong recurrent interactions that support sustained activity patterns and could be more vulnerable to hyperexcitability in LOF variants,^51^ whereas feed-forward thalamocortical circuits might be affected preferentially in GOF variants. Other mechanisms have been proposed for GOF variants, including reversal of chloride gradients and increased GABA-mediated excitation in developing neurons that delay or impair normal brain development.^2,3,11^ Developmental timing is therefore likely to be critical, because early alterations in GABAergic signalling can disrupt circuit maturation and produce lasting network instability.^52-54^ These mechanisms have not been detected in β3^E77K^ mice. However, because recordings were conducted in adult animals, early developmental dysfunction would not have been captured. More severe variants induced lethality, and we cannot rule out the potential contribution of these mechanisms to part of the GOF clinical spectrum, particularly when the clinical outcomes are broad and multi-systemic.

## Conclusion

GOF is observed in ∼40% of pathogenic variants in *GABRB3*-related disease.^2^ Unfortunately, treatment options for those impacted by these variants are limited, and the mechanisms driving neuronal phenotypes in GOF disease remain poorly understood. In this study, we demonstrate that the β3^E77K^ GOF mouse model exhibits unique behavioural characteristics when compared with mouse models with LOF GABA_A_ receptor variants. These traits align with the stratification observed in patients.^2,14,15^ A significant discovery is the heightened interneuron-to-interneuron GABA_A_ receptor-mediated synaptic currents, which are likely to fuel the activity-dependent reduction in feed-forward inhibition observed in layer 2/3 of β3^E77K^ mice. Overall, β3^E77K^ mice provide new models that allows us to decipher the intricate cellular and neuronal network dysfunctions caused by GOF GABA_A_ receptor variants that might also have relevance to other monogenic epilepsies.

## Supplementary Material

awag119_Supplementary_Data

## Data Availability

Raw data and images are available upon request from the corresponding author.
