# Supplementary material for: Collapse of feed-forward inhibition underpins hyperexcitability in GABAA receptor gain-of-function epilepsy
Source: Brain. 2026 Apr 22;149(11):3789–805. doi: 10.1093/brain/awag119 (PMC13730555; doi:10.1093/brain/awag119)
Supplement: Supplementary file 1 [file awag119_supplementary_data.pdf]

Supplementary Methods

Generation of the β3^{E77K} and other Gabrb3 mouse models

Knock-in mouse models were generated using CRISPR-based technologies in a C57Bl/6J background by the Monash Gene Modification Platform (MGMP) (Supplementary Figure 5, Supplementary Table 4). Briefly, Cas9 nuclease (30 ng/ml), sgRNA (30 ng/ml) & repair templates 1 & 2 (30 ng/ml) were microinjected into the pronucleus and cytoplasm of C57Bl/6J zygotes at the pronuclei stage (sequences in Supplementary Table 5). A wild-type repair oligo was also used to decrease the probability of founder mice being created on null or homozygous backgrounds. Injected zygotes were transferred into the uterus of pseudo pregnant F1 females. The offspring were genotyped using PCR primers and restriction enzymes (Supplementary Table 5). Founder mice positive for the knock-in were confirmed by sequencing for the point mutation and then bred on a C57Bl/6J background. Lines were discontinued when viable offspring containing the knock-in variant were not identified after a minimum of 4 litters. For β3^{E77K} line, the mutation was subsequently backcrossed for at least one generation into a C57Bl/6N background.

Transcardial perfusions and immunohistochemistry

Mice (aged 12-20 weeks) were anaesthetised and perfused with 4% paraformaldehyde in PBS. Brains were extracted and post-fixed overnight in 4% paraformaldehyde in PBS, then washed with PBS and stored in PBS at 4 °C. 50 µm-thick coronal brain sections were cut with a cryostat and free-floating sections were used for immunofluorescence analysis.

Tissue Preparation

Tissues were fixed in 4% paraformaldehyde (PFA) in PBS and cryoprotected by increasing sucrose concentration in PBS before cryosectioning at 30 µM sections using a cryostat (Leica

CM1950). Sections were collected in a 1:10 series and stored in antifreeze (containing 50% 0.1 M sterile PBS/30% ethylene glycol/20% glycerol) at -20 °C until required. Unless specified, tissues were washed 3 times for 10 minutes with PBS for all immunohistochemical processing. Experiments were performed on free-floating sections; all washes, incubations, and reactions took place in a glass vial on an orbital shaker at 100 rpm.

Nissl staining and analysis

Briefly, sections were mounted on gelatinised slides and air-dried prior to staining. Slides with mounted sections were washed briefly in tap water and dehydrated by passing through increasing concentrations of ethanol (70%, 95%, 100%), then rehydrated with lowering the concentrations of ethanol (95%, 70%) for 2-3 minutes each. Slides were stained with 0.1% cresyl violet for 8 minutes, washed briefly with distilled water and 20 s washes with 50%, 70%, and 100% ethanol. Slides were then cleared in xylene for 10 minutes and coverslipped with Eukitt. Imaging for Nissl stain was conducted using Leica Aperio at 20X objective.

Volume estimation

Staining the tissue with cresyl violet allowed for the delineation of brain regions. The size of the hippocampus was measured by tracing around the hippocampus and then corrected for the thickness and series of tissues collected. The structural volume of the brain (from -1.94 mm - -2.54 mm of bregma) was calculated using the Cavalieri method.[1] Such that:

$$Hippocampus\ volume\ (mm^3) = (hippocampus\ area) \times (tissue\ thickness) \times (number\ of\ sections)$$

where, each section is 30 um thick, and one section out of 10 was used for cresyl violet staining.

FluoroJade C

Fluorojade C (FJC) is a polyanionic fluorescin derivative that exhibits a high degree of sensitivity that selectively binds to degenerative neurons, allowing for detection of apoptotic cells from early to late stages of apoptosis.[2,3] FJC stains for neuronal death unspecific to the death pathway, which is beneficial to scope whether neuronal death occurs in GOF mice. Briefly, sections were washed in PBS and then pre-treated with ethanol to rehydrate the tissues and allow for permeabilisation. Tissues were placed in 80% ethanol with 1% sodium hydroxide, followed by 70%, 50%, 30% and 10% ethanol and then passed in water. To reduce fluorescent background, sections were then placed in 0.06% potassium permanganate solution, a fluorescence quencher for 10 minutes, washed in ddH_20 and immersed in 0.0001% fluoro jade C with DAPI (1:1000) for 20 minutes and then washed 3 times in ddH_2O. Brain sections were then recovered in PBS, mounted onto 2% gelatinised sides, and sealed with fluoroshield (Sigma-Aldrich). Images were scanned with Leica DM6000 at 20X objective.

Iba-1 staining and analysis

Iba-1 is a marker expressed on microglia, the resident immune cells of the brain. Immunohistochemistry was performed as previously described.[4] Briefly, free-floating sections were removed from the anti-freeze solution and washed in PBS. To allow for cell permeabilisation, sections were washed in 50% ethanol for 30 minutes. Sections were then treated in 3% H_2O_2 in 50% ethanol to quench endogenous peroxidase activity. Wash with PBS ensued, and the sections were placed in a blocking solution of 10% normal horse serum (NHS) in 0.1 M PBS for 30 minutes and then incubated in iba-1 (1:1000 in 2% NHS/PBS) at 4°C overnight. Sections were then washed and incubated in horse anti-rabbit IgG (1:500 in 2% NHS in PBS) for 2 hours, washed, and incubated in ExtrAvidin Peroxidase (1:1000 in PBS) for 2 hours. Iba-1 was then visualised with 3,3'-diaminobenzidine (DAB) kit as per the

manufacturer's instruction. The reaction ran for 10 minutes and stopped by washing the section 4 times in PBS.

Sections were then mounted onto gelatinised slides, dehydrated, and defatted using xylene prior to cover slipping with Eukitt. Images were scanned with Leica Aperio at 20X objective. Photomicrographs of 3-5 sections of the brain, between -1.94 to -2.54 mm of bregma, were collected through data analysis. Results are reported as the average number of iba-1 positive cells per section for each mouse.

Immunohistochemistry fluorescence

Briefly, sections were thoroughly washed in PBS. To minimise fixative-induced autofluorescence, sections were treated with 0.1% sodium borohydrite for 10 minutes. To allow for membrane permeabilisation, sections were washed in 0.3% TritonX in PBS and blocked with 10% normal goat serum (NGS) in 0.3% triton-X in PBS for 1 hour. Sections were then incubated in anti-MBP (1:1000), anti-Znt3 (1:500), in 5% NGS/0.15% triton-X/PBS overnight at 4°C. Sections were then washed thoroughly in PBS at room temperature and incubated with AlexaFluor 568 secondary antibody (1:500) in 5% NGS in 0.15% triton X in PBS for 1 hour. Once the secondary antibody was applied, sections were protected from light to prevent the loss of fluorophores. Cryosections were then washed and incubated in DAPI for 30 minutes, then mounted onto gelatinised slides and cover slipped with fluoroshield (Sigma-Aldrich).

**Antigen**		**Host**	**Source, catalog #**	**Concentration IHC**
Anti-Myelin basic protein	Primary	Rabbit	Abcam, AB40390-1001	1:1000
ZnT3	Primary	Rabbit	Focus Bioscience, 197 003	1:500
Alexa-Fluor 568	Secondary	Goat		1:500

Fluorescence imaging of myelin basic protein was performed using an Olympus 120 VS with a 20X objective, and mossy fibre imaging was performed using a DM6000 with a 20X objective.

Immunofluorescence staining of biocytin-filled neurons and parvalbumin-positive interneurons (300 µm vibratome sections)

Following whole-cell recordings with internal solution containing 0.2% biocytin, neurons were processed for post hoc immunofluorescence to assess cellular morphology and, where feasible, parvalbumin (PV) expression. PV immunostaining was successfully performed in a subset of recorded interneurons (including cells from mIPSC and intrinsic firing datasets), allowing direct confirmation of PV expression. For recordings where PV verification was not obtained, interneuron identity was defined using convergent anatomical (including laminar position in layer 2/3, basket-cell morphology) and electrophysiological criteria (including membrane capacitance <80 pF and fast-spiking firing properties where measurable). PV-verified interneurons did not differ in intrinsic or synaptic properties from the broader physiologically identified interneuron population, and data were therefore pooled.

Acute brain slices (300 µm) were immersion-fixed in freshly prepared 4% paraformaldehyde (PFA) in 0.1 M phosphate buffer (PB; pH 7.4) for 2-4 h at 4 °C (short fixation to preserve antigenicity). Slices were rinsed in PBS and stored up to 7 days at 4 °C in PBS containing 1% penicillin-streptomycin (or 0.02-0.05% sodium azide for extended storage).

Free-floating slices were washed 3 × 10 min in PBS (RT, rocking) and blocked overnight at 4 °C in PBS containing 10% normal goat serum (NGS), 0.3% Triton X-100, and 0.03% ProClin (to prevent microbial growth during multi-day incubations).

Slices were incubated for 5 days at 4 °C (gentle rocking, sealed, light-protected) in primary diluent (PBS + 2% NGS, 0.3% Triton X-100, 0.03% ProClin) containing rabbit anti-parvalbumin (1:2000). Aliquoted antibody was stored at -80 °C to avoid repeated freeze-thaw. Slices were then washed 3 × 15 min in PBST (PBS + 0.1% Triton X-100) at RT, then PBS rinse.

Slices were incubated overnight at 4 °C (dark) in secondary diluent (PBS + 2% NGS, 0.1-0.3% Triton X-100, 0.03% ProClin) containing streptavidin-Alexa Fluor 594 (1:1000) to visualise biocytin-filled neurons and goat anti-mouse IgG2a-HRP (1:1000) when applicable. (Fluorescent incubations were protected from light).

After 3 × 15 min PBST and a final PBS rinse, slices were incubated in DAPI (1 µg/mL) for 30 min (RT), washed in PBS, then mounted in aqueous antifade medium (ProLong Glass or Fluoromount-G). Coverslips were sealed with clear nail polish and cured overnight (RT, dark). Confocal images were acquired within 24-48 h (10× - 40× objectives) with a Zeiss LSM780 confocal microscope. Laser power, detector gain/offset, pixel size, and z-step were held constant across genotypes and conditions. Biocytin was visualised with streptavidin-AF594 conjugate (red), PV with L114/3 immunolabelling (green), and nuclei with DAPI (blue).

**Antigen**		**Host**	**Source, catalog #**	**Concentration IHC**
Anti-Parvalbumin	Primary	Mouse	DSHB, L114/3	1:2000
Goat Anti-mouse IgG2a	Secondary	Goat	Abcam, ab97245	1:1000
Streptavidin AF594 conjugate		N/A	Invitrogen, S-32356	1:1000
DAPI		N/A	Sigma Aldrich, D9452	1 µg/mL

Electrocorticography (ECoG)

ECoG electrode implantation surgery

Male and female β3^{E77K} mice and wild-type littermates, 6-16 weeks of age, were used for ECoG experiments. Mice were anaesthetised via isoflurane inhalation (4% for induction, 1-2% for maintenance throughout surgery), placed on a stereotaxic frame, and administered meloxicam (2.5 mg, subcutaneous, Ilium) as an analgesic and lidocaine hydrochloride (10 mg, subcutaneous, Pfizer) to provide local anaesthesia to the scalp. An incision was made and three holes, each 1 mm in diameter, were drilled into the skull. Two of these holes were drilled bilaterally over the somatosensory cortex, with the third located immediately caudal to the

lambdoid suture 0.5 mm lateral from the midline towards the right side of the skull. Stainless steel screw electrodes were implanted into each hole, with the anterior two screws implanted epidurally and used as the active channel electrodes, and the posterior screw implanted slightly more shallowly and used as the reference channel electrode. A ground electrode made of silver wire was affixed to the skull immediately caudal to the lambdoid suture 0.5 mm lateral from the midline towards the left side of the skull. The reference, ground, and two active channel electrodes were connected to a mouse electroencephalography (EEG) head mount (8201-EEG Pinnacle technology Inc.) via silver leads (Cat No. 785500, A-M Systems Inc.) and soldered in place. Self-curing acrylic resin was used to hold the head mount and electrodes in place. Mice were left to recover for at least one week prior to experimentation.

ECoG recordings

Brain cortical activity was sampled at 250 Hz (Pinnacle Technology), with a low pass and high pass filter of 40 Hz and 0.5 Hz, respectively. During recording, mice could move freely in the cage with food and water *ad libitum*. Continuous 24-hour recordings were conducted to characterise baseline ECoG activity during the light and dark phases.

ECoG data were analysed by a blinded experimenter using Sirenia Seizure software (Pinnacle Technology). Power spectra were generated using the program's built-in FFT-based spectral analysis and expressed as absolute power ($\mu V^2/Hz$). For each animal, spectral power was averaged across 30-min epochs sampled at fixed time points within the light and dark phases to enable between-animal comparisons. Mice were excluded from spectral analysis if any noise (electrical noise or movement artifacts) was present in the recording trace or if a momentary loss of signal confounded the data.

Spike-wave-like bursts were identified and quantified manually by a blinded experimenter based on defined morphological and temporal criteria. Events were required to consist of rhythmic spike-and-wave discharges comprising ≥3 consecutive cycles at 7-10 Hz, lasting 0.3-2.0 s, and visible across both cortical channels. Events were distinguished from sleep spindles by their lower frequency, abrupt onset and offset, and distinct waveform characteristics. Simultaneous video monitoring did not reveal consistent stereotypic behaviours or behavioural arrest during these events, and they were therefore classified as spike-wave-like discharges rather than absence seizures. Recordings were excluded from analysis if electrical noise, movement artefacts, or transient signal loss were detected.

Drug testing experiments

To assess drug effects on cortical spike-wave-like activity, separate cohorts of $\beta3^{E77K}$-6J mice were implanted with ECoG electrodes and recorded continuously during baseline and following drug administration. Vigabatrin (300 mg/kg, intraperitoneal; Sigma-Aldrich) or its vehicle (0.9% saline) was administered after a 2-h baseline period, and recordings continued for a further 2 h. Valproate (200 mg/kg, intraperitoneal; Sigma-Aldrich) was tested using a 1-h pre-injection baseline and a 1-h post-injection recording window due to its shorter half-life in mice. Spike-wave-like discharges were manually quantified by a blinded experimenter using the same morphological and temporal criteria described above. The number of events was compared between pre- and post-injection periods within each animal.

Behavioural Assays

Mice were allowed to acclimatise in their home cages to the behavioural room for at least 1 hour prior to the commencement of any behavioural assays.

Open-field locomotor assay and thigmotaxis

Mice (aged 3-12 weeks) were placed in a square 27.3x27.3x20.3 cm open-field arena and were allowed to move freely for one hour, during which infrared rays tracked their movement. Data indicating distance travelled and other parameters were recorded and compiled using MED Associates Activity Monitor software (Med Associates Inc.). To study thigmotaxis, time spent in a 19.05x19.05 cm centre zone of the open-field arena was compared to time spent in the outer perimeter of the arena.

Light-dark box assay

A square mouse open field arena was divided into two zones of equal size (27.3x13.7 cm). One half of each arena was illuminated at 750 ± 25 lux using a small lamp (the 'light' zone), while the other half was enclosed with a black acrylic box insert with a lid and a small opening on one side (the 'dark' zone). Mice (aged 12 -20 weeks) were individually placed in the dark zone and allowed to freely explore both zones for 10 minutes. Data indicating the duration of time spent in each zone was recorded and compiled using MED Associates Activity Monitor software (Med Associates Inc.).

Rotarod assay

Mice (aged 12-20 weeks) were trained to walk on the rotarod over three sessions: two 2-minute sessions at a fixed speed of 4 rotations per minute (rpm), and one 2-minute session accelerating from 4-20 rpm. Mice were then tested on the rotarod for three 5-minute trials (with at least 30 minutes between trials). Each trial consisted of 5 minutes where the rotarod accelerated from 4-40 rpm. Latency to fall was recorded.

Ledged beam assay

Motor coordination and balance were assessed using the ledged beam test as described previously.[5] The ledged beam apparatus consisted of a 100-cm-long wooden beam tapering from 8 cm to 1.5 cm in width, positioned at a 15° incline approximately 90 cm above the floor. Underhanging ledges (0.5 cm wide) were fixed 2 cm below the upper surface on both sides of the beam to allow animals to step down if they lost balance. Four towels were placed beneath the beam to cushion potential falls.

Male and female $\beta 3^{E77K}$-6J and wild-type littermate mice (aged 12-20 weeks) were trained over five trials to traverse the beam from the wide (lower) end to the narrow (upper) end, which led directly into the home cage. Each trial lasted <1 minute; if a mouse failed to cross within 10 minutes, the trial was terminated and the animal excluded from further analysis.

Testing was performed after training, with three consecutive trials recorded by a ceiling-mounted video camera. Videos were scored offline by a blinded observer. A foot fault was defined as a step onto the lower ledge, and the total number of foot faults for each limb was expressed as a percentage of total steps taken to traverse the beam. The beam was also divided into three equal sections (wide, middle, narrow) to assess performance across increasing levels of difficulty.

Grip strength assay

Grip strength testing was performed using a digital grip strength meter (Able Scientific, Australia) following the manufacturer's standard procedure. The apparatus was placed on a stable laboratory bench under normal lighting conditions similar to the holding room. The unit was set to measure force in grams (g) using the grid or bar attachment secured by the locking screw.

Male and female β3^{E77K}-6J and wild-type littermate mice (aged 12-20 weeks) were tested individually. Each mouse was gently held by the base of the tail and lowered toward the attachment until its forepaws grasped the grid. The mouse was then brought to an approximately horizontal position and pulled steadily backward in line with the attachment rod until it released its grip. The maximum force (in grams) displayed by the meter was recorded as the peak grip strength.

Each mouse completed five consecutive measurements separated by 30-second rest periods. The mean peak force per animal was used for statistical analysis. The grid surface was wiped with ethanol between animals to maintain hygiene and prevent scent cues.

Clasping behaviour

After a 30-minute acclimatization period in the testing environment, the animals underwent tail suspension for a minimum of 10 seconds, with all sessions being video recorded. Hindlimb clasping behavior was evaluated using a grading scale ranging from 0 to 3, following the criteria established by Guyenet et al. (2010)[6], where:

- 0: Both hindlimbs extended outward away from the abdomen.
- 1: One hindlimb withdrew towards the abdomen for at least 50% of the observation period.
- 2: Both hindlimbs withdrew towards the abdomen for at least 50% of the observation period.
- 3: Both hindlimbs remained withdrawn, making contact with the abdomen.

This testing procedure was repeated three times across three consecutive days. Two independent observers, blinded to the experimental conditions, scored the video recordings, and the average score for each animal was documented.

Elevated plus maze

The elevated plus maze consists of a plus-shaped platform raised 40 cm from the floor. Two of the arms, on opposite sides, are enclosed with 55 cm high walls extending from the centre, with the other two arms remaining open with no walls. Mice (aged 12-20 weeks) were individually placed in the centre of the maze facing one of the open arms and were allowed to freely roam the maze for 10 minutes, and their movements were tracked by a ceiling-mounted video camera. Entries into each arm, along with their respective durations, were measured using tracking software (CleverSys TopScan Lite).

Y-maze

The Y maze consists of a Y-shaped Perspex enclosure with three 7.5x30x14 cm arms each containing a distinct visual cue at the end. The test was performed in two phases: a training session and a test session, with a one-hour interval in between. During the training session, access to one of the three arms (the 'novel' arm) was blocked by an opaque Perspex barrier. Mice (aged 12-25 weeks) were allowed to explore the two accessible arms (the 'home' arm – where they are first placed – and the 'familiar' arm) of the maze for 10 minutes. During the test session, the barrier blocking the novel arm was removed and mice were placed into the home arm and allowed to explore all three arms for 5 minutes. The time the mouse spent in each of the three arms was measured using tracking software (CleverSys TopScan Lite).

Three-chamber social interaction assay

This test was conducted in a 43x39x11 cm enclosure made of transparent plastic and divided into three chambers linked by small rectangular openings that mice could freely move through. Metal mesh cages (16x10x11 cm) were placed at one end of each of the two lateral chambers. The assay consisted of two trials, habituation and test, which occurred successively. During the habituation phase, mice (aged 12-25 weeks) were individually placed into the central chamber

and allowed to freely explore all three chambers for 10 minutes. Their movements were tracked using a ceiling-mounted video camera and measured using tracking software (CleverSys TopScan Lite). The side which each mouse preferentially spent more time was noted. During the test phase, an unfamiliar age- and sex-matched 'stranger' mouse was placed into the mesh cage on the opposite side to the test mouse's preference. The test mouse was then returned to the enclosure and allowed to explore all three chambers for 10 minutes. Time spent interacting with the stranger mouse was quantified using tracking software (CleverSys TopScan Lite).

Barnes maze

The Barnes maze comprises a large circular white Perspex arena (1.2 m diameter) raised 56 cm off the ground, with 36 holes evenly spaced around the outside. One hole was the 'target', under which was a small, dark escape box, which mice could freely enter. The maze was arranged with visual cues on surrounding walls and was brightly lit using floodlights. On Day 1, mice (aged 12-25 weeks) were familiarised to the escape box by being individually placed inside it for 5 minutes. The assay took place over seven consecutive days. Mice performed four trials per day, with 20-30 minutes between each trial. At the start of a trial, the floodlights were turned on and mice were individually placed in the centre of the maze underneath a darkened cover. The cover was lifted via pulley, and mice were able to freely explore the maze for a maximum of 4 minutes. Their movements were tracked using a ceiling-mounted video camera and measured using tracking software (CleverSys TopScan Lite). Trials ended if the mouse entered the escape box or if the 4 minutes elapsed, at which point the mouse would be guided into the escape box. At the end of a trial, the floodlights were turned off and the mouse left to rest in the escape box for 30 seconds before being placed back into its home cage. Average latency to first locating the target hole was calculated (excluding trials where the mouse jumped or fell off the maze). Mice performed four trials per day for 5 consecutive days. 24 h after the

last acquisition trial, a reversal trial, in which the location of the 'target' hole was reversed, was performed.

Two-electrode voltage-clamp experiments

Two-electrode voltage-clamp electrophysiology was performed using *Xenopus* laevis oocytes as described previously.[7] Briefly, fully concatenated wild-type or variant (single $\beta3^{E77K}$ or double $\beta3^{E77K}$) γ2-β3-α1-β3-α1 cDNA were created using our established concatenation strategy[8]. Twenty-five ng cRNA of the transcribed cDNA were injected into stage V-VI oocytes and incubated for 2-3 days at 18 degrees prior to functional testing. On the day of recording, oocytes were placed in a custom-built recording chamber continuously perfused with ND96 (96 mM NaCl, 2 mM KCl, 1 mM $MgCl_2$, 1.8 mM $CaCl_2$ and 10 mM HEPES at pH 7.4). To quantify GABA sensitivity (EC_{50}), concentration-response relationships were obtained by applying increasing GABA concentrations to the oocytes with 2-5 minutes washout in between applications. Maximum GABA-evoked current amplitudes (I_{max}) were measured by applying a saturating 10 mM concentration of GABA to the oocytes. To determine desensitisation properties, current decay rates (k) and steady-state currents (I_{ss}/I_{peak}) were assessed by applying 3 mM GABA to the oocytes for 150 s.[9]

Data analysis

Analyses were performed using GraphPad Prism 10 (Dotmatrics, USA). The logarithmic of EC_{50} was computed to calculate the change in $LogEC_{50}$ ($\Delta LogEC_{50}$) compared to the daily average wild-type value. Statistical significance was determined using one-way ANOVA with Dunnett's post hoc test. Normalised I_{max} values were compared using Mann-Whitney U test. Desensitisation properties, k and I_{ss}/I_{peak}, were compared using non-parametric one-way ANOVA (Kruskal Wallis rank sum test) with Dunn's post hoc test. For all statistical analysis, a threshold of $P < 0.0001$ is used for statistical significance.

Brain slice electrophysiology

Slice preparation

For whole-cell recordings, brain slices were prepared from P20-34 day old mice while 6-12 week old mice were used for feed-forward inhibition experiments. Briefly, the mouse was anesthetized with isoflurane in a transparent chamber until the righting reflex was abolished, then removed and rapidly decapitated. The brain was extracted and put into ice-cold slicing solution, containing (in mM) 87 NaCl, 3 KCl, 0.25 $CaCl_2$, 3 $MgCl_2$, 25 $NaHCO_3$, 1.25 NaH_2PO_4, 75 sucrose, and 25 glucose (305mOs/kg) for whole-cell recordings or in 200 Sucrose, 10 D-glucose monohydrate, 26 $NaHCO_3$, 1.25 NaH_2PO_4, 2.5 KCl, 0.1 $CaCl_2$ dihydrate, 0.5 L-ascorbic acid, 2 Na-pyruvate, 3 myo-inositol/mannitol, 4 $MgCl_2$ for feed-forward inhibition recordings. The brain was then fixed with superglue onto the slicing chamber and 300 µm-thick sagittal (feed-forward inhibition) or coronal (everything else) brain slices were prepared using a vibratome (Leica VT1200S). Approximately 4-5 slices were collected from each brain. The slices were then transferred to a holding chamber containing artificial cerebrospinal fluid (ACSF) comprising (in mM) 125 NaCl, 3 KCl, 2 $CaCl_2$, 1 $MgCl_2$,

25 $NaHCO_3$, 1.25 KH_2PO_4, and 25 glucose (310mOs/kg), supplemented with 3.6 mM pyruvate and 1.7 mM ascorbate for whole-cell recordings or in ACSF containing 111 NaCl, 25 $NaHCO_3$, 1.1 NaH_2PO_4, 3 KCl, 2.5 $CaCl_2$, 1.3 $MgCl_2$, and 11 glucose for feed-forward inhibition recordings. Both the slicing solution and ACSF were continuously bubbled with carbogen (95% O2/5% CO2). The holding chamber was initially kept at 28-32 °C for 30-60 min, then allowed to cool to room temperature to maintain the slices until required.

Electrophysiology for whole-cell recordings

Whole-cell recordings were made in current clamp mode to measure firing properties or in voltage-clamp mode to measure the miniature Postsynaptic Inhibitory Currents (mIPSCs). Briefly, a slice was taken from the holding chamber and put into the recording chamber, which was continuously perfused with carbogen-bubbled ACSF. The temperature of the perfusion solution was kept at 32-34 °C using a feedback-controlled inline heater (Warner Instrument, USA) and the flow rate was maintained at ~3-4 mL min^{-1} throughout the experiment. The slice was secured in place using a 'harp' made of a flattened U-shaped platinum wire across which was stretched 4-6 single strands of nylon dental floss. The cells of interest were visually identified by using an Olympus BX51WI microscope with a TH4-200 controller (Olympus, Tokyo, Japan). Whole-cell patch clamp recordings were made from CA1 pyramidal neurons, CA1 interneurons, layer 2/3 cortical pyramidal neurons and layer 2/3 cortical interneurons. For the current clamp and sEPSC recordings, the internal solution contained (in mM) 125 K-gluconate, 5 KCl, 2 $MgCl_2 \cdot 6\ H_2O$, 10 HEPES (4-[2-hydroxyethyl]-1-piperazine-ethane sulfonic acid), 0.1 EGTA (ethylene glycol-bis[β-aminoethyl ether]-N,N,N′,N′-tetraacetic acid), 10 phosphocreatine Na_2, 4 $Mg_2 \cdot ATP$, 0.3 $Na_3 \cdot GTP$ and 0.2% biocytin (290-300 mOs/kg, pH 7.2, liquid junction potential (LJP) = 15.1 mV) was filled into the recording electrode. For the voltage-clamp recordings for mIPSCs, the internal solution containing (in mM) 135 KCl, 7 NaCl, 10 HEPES, 0.1 EGTA, 2 $MgCl_2$, 2 $Na_2 \cdot ATP$, 0.3 $Na_3 \cdot GTP$, 5 QX-314·Cl and 0.2%

biocytin (290-300 mOs/kg, pH 7.2, LJP = 3.7 mV, equilibrium potential of Cl^- = 151 mV) was used. The identified cell was then approached with a strong positive pressure (~40 millibars) and the pipette offset was adjusted and the electrode resistance was confirmed to be in the range 4-8 MΩ. The electrode was then approached to the cell of interest. After contact with the soma membrane, the positive pressure was released, and gentle suction applied. Upon achieving a GΩ seal, a -70 mV holding potential was applied. Bursts of gentle suction were used to break into the cell and achieve stable access. Cell types were identified firstly by their location. For example, in the CA1 region of the hippocampus, pyramidal cells were recorded in the pyramidal cell (PC) layer, while interneurons were recorded exclusively from the stratum oriens (SO) layer. A subset of the cells were recorded using normal internal solution to identify the firing properties and cell membrane capacitance of the specific cell type (e.g. the interneurons in SO layer have fast spiking pattern with lower cell membrane capacitance compared to the pyramidal cells in PC layer). Interneuron inclusion criteria were: small, rounded somatic profile, fast-spiking firing pattern on depolarizing steps (assessed with normal internal solution), and membrane capacitance <80 pF. For interneurons in the CA1 hippocampus, inclusion criteria also included soma in stratum oriens. Additionally, the cells were filled with 0.2% biocytin via the internal solution during whole-cell recordings to enable post hoc morphological reconstruction and, where applicable, subsequent immunohistochemical analysis (details above). Normal ACSF (mentioned above) was used as the external solution for current clamp recordings while in voltage-clamp recordings of mIPSCs, the ACSF was supplemented with 10 µM CNQX, 20 µM DL-AP5 and 0.5 µM TTX to block AMPA receptors, NMDA receptors and voltage-gated sodium channels respectively. In some of the experiments, the external ACSF was switched to the one containing 100 µM picrotoxin to confirm the isolation of GABA-mediated mIPSCs in voltage-clamp recordings.

The recordings were made using Axon Multiclamp 700B amplifier, Digidata 1440A digitizer (Molecular Devices), and pCLAMP version 10 software (Molecular Devices, USA). For current clamp recordings, Multiclamp was switched to current clamp mode after getting stable access. Cells were typically allowed to remain at their resting membrane potential. Before recording, bridge balance and capacitance neutralisation were adjusted carefully and checked for stability. A family of 1000 ms-long current steps were then applied, with increments of 10 pA (total 30 steps, repeated every 5 sec). For voltage-clamp recordings, after gaining stable access, the series resistance compensation was done (60-75%) in the whole-cell mode followed by the recording of mIPSCs at the holding potential of -70 mV for 3 min.

Electrophysiology for feed-forward inhibition experiments

Brain slices were carefully positioned within a recording chamber beneath the objective lens of an upright microscope equipped with epifluorescence illumination (SliceScope Pro 1000, Scientifica Ltd, United Kingdom or Olympus BX51). The chamber maintained continuous perfusion of ACSF saturated with carbogen to ensure optimal slice viability. Patch electrodes, with a resistance ranging from 4 to 7 MΩ, were crafted using a P-1000 pipette puller (Sutter Instruments Co., USA). Recordings of pyramidal neurons located in layer 2/3 of the somatosensory cortex were performed using the whole-cell patch clamp configuration, either in voltage or current-clamp mode. For voltage-clamp recordings, pipettes were filled with a solution containing (in mM) 108 Cesium gluconate, 5 tetraethylammonium-Cl, 2.8 NaCl, 20 HEPES, 0.4 EGTA, 4 MgATP (all from Sigma-Aldrich), 0.3 Na_2GTP (Roche), 1 QX-314 (Tocris); LJP = 8.0 mV. For current-clamp recordings, pipettes were filled with a solution comprising (in mM) 108 K-gluconate, 8 KCl, 8 Na-gluconate, 1 EGTA, 10 HEPES, 2 $MgCl_2$ hexahydrate, 4 K_2ATP (all from Sigma-Aldrich), 0.3 Na_2GTP (Roche); LJP = 14.6 mV. Precise positioning of the patch pipettes was facilitated by a three-axis motorised micromanipulator (PatchStar, Scientifica Ltd, United Kingdom or Luigs and Neumann,

Germany), connected to a CV-7B Headstage (Molecular Devices, Sunnyvale CA, USA). Recordings were conducted utilizing a MultiClamp 700B amplifier (Molecular Devices, CA, USA). Data were sampled at a rate of 20 kHz and transmitted to a computer via a Digidata 1550B digitizer (Molecular Devices, Sunnyvale CA, USA). Synaptic currents in pyramidal neurons from layer 2/3 of the somatosensory cortex were recorded from $\beta 3^{E77K}$ animals and wild-type littermates. Throughout the recordings, access resistance was regularly monitored. Data points were excluded if the access resistance exceeded 30 MΩ or if it fluctuated by more than 20% during the recording session. Pyramidal cells were also excluded from analysis if their membrane potential surpassed -55 mV.

A bipolar stimulation electrode (TM33CCNON; World Precision Instruments, Sarasota, FL, USA), coupled with a stimulus isolator (A365RC, WPI, UK), was carefully positioned within layer 4 of the somatosensory cortex. The threshold for triggering synaptic responses (Th EPSC) was meticulously calibrated to evoke consistent EPSCs (5 EPSCs out of 5 consecutive trials) in the recorded pyramidal cell.

For dual stimulation, two electrodes were placed in layer 4, flanking either side of the recorded pyramidal cell in layer 2/3. The separation distance between the stimulation electrodes varied within a range of 200 to 300 µm. To assess excitability, the firing probability was measured relative to the minimal intensity required to evoke 5/5 action potentials (Th AP) in the pyramidal neurons. Conditioning trains consisting of 10 shocks at 10 Hz were administered at 1.2 times the minimal intensity needed to elicit 5/5 EPSCs. The membrane potential was measured immediately before and after the conditioning train to confirm that the conditioning did not alter the resting potential (WT: -69.0 ± 3.6 mV vs -69.2 ± 2.9 mV, p = 0.9; E77K: -66.0 ± 1.9 mV vs -65.7 ± 2.0 mV, p = 0.78; n = 7 WT, 7 E77K; Wilcoxon signed-rank test).

Data Analysis

Input resistance (R_{in}) was calculated by measuring membrane potential (V_m) near the end of the two hyperpolarizing current steps (I_{cmd}); the slope of the V_m vs I_{cmd} plot was then calculated to yield R_{in}. Resting membrane potential (RMP) was defined as the mean Vm measured over a 50 ms window immediately before the start of the current step. Rheobase was defined as the amplitude of the smallest 1 s long current step that elicited at least one action potential (AP). Action potential firing frequency was counted for all current steps and used to plot f-I curves (frequency vs current relationships). LJP was calculated for each pair of internal-external solutions (see above) and not corrected in reported V_m values.

The IPSCs are detected using Axograph. A template was created to isolate the IPSCs (standard deviation of 4 - 4.5) followed by manual sorting to remove any which did not appear to be true IPSCs. The peak amplitude was the peak amplitude of the IPCSs calculated from the baseline. Halfwidth was defined as the width of the IPSCs halfway between the baseline and the peak. Rise time was the time from 10% to 90% of the IPSCs amplitude. Decay time was the time from 100% to 50% of the IPSCs amplitude. The area was calculated from the area under the curve of the isolated IPSCs. The frequency was also calculated from the isolated traces. For the tonic current measurement, the baseline mean current was obtained by fitting a Gaussian to the all-point histogram over 1 s epochs and compared between the baseline shift before and after the application of 100 μM picrotoxin.

Behavioural data reporting. No animals were excluded from behavioural analyses. Differences in group sizes reflect the use of separate littermate cohorts across experimental paradigms (not attrition). Group sizes (n) are reported in figure legends. Allocation to genotype was by Mendelian outcome; experimenters were blinded to genotype during acquisition and analysis wherever feasible. Note that, as stated elsewhere, occasional data points (but not animals) were excluded when >2 SD from the group mean per a priori criteria.

RNAseq dataset

The mouse whole cortex and hippocampus single cell RNAseq dataset[10] was available from the Allen Brain Map portal (https://portal.brain-map.org/atlases-and-data/rnaseq/mouse-whole-cortex-and-hippocampus-smart-seq). The dataset, metadata and processed tSNE (t-distributed Stochastic Neighbour Embedding) coordinates were imported into *Seurat* (v4.3) for analysis. The primary somatosensory region (SSp) was subset using the *subset* function, followed by data normalisation by variance stabilising transformation using *SCTransform* (v2). Manual cell annotation was performed to categorise primary somatosensory region cells using the following marker genes: all interneurons (*Gad1*+), PV positive interneurons (*Gad1*+ *Pvalb*+), pyramidal cells (*Cux2+)*. All other cells are labelled as 'Others'. Visualisation of gene expression was generated using the *Vlnplot* function.

Feed-forward network model

To examine the impact of $GABA_A$ receptor GOF on cortical feed-forward inhibition, we created a network model containing an excitatory pyramidal cell (PC) and 50 inhibitory basket cells (BC). Cell and synapse models were based on Bryson et al., 2021[11] and BC-to-PC synapses were modified to include a slow component as observed in Dos Santos et al., 2024[12]. BC-to-BC and BC-to-PC connection probability was set to 1 and conditioning/test stimuli comprised poisson-distributed synaptic input lasting 10 ms. The test stimulus was set as the minimum frequency that consistently elicited spikes from the PC population, and the condition stimulus was set at one-third of this value. *GABRB3* GOF was modelled by increasing the peak BC-to-BC synaptic conductance. Simulations were performed using NetPyNE[13] and Neuron[14].

References

1. Wyse ATS, Sanches EF, Dos Santos TM, Siebert C, Kolling J, Netto CA. Chronic mild hyperhomocysteinemia induces anxiety-like symptoms, aversive memory deficits and hippocampus atrophy in adult rats: New insights into physiopathological mechanisms. *Brain Research.* 2020;1728:146592.
2. Schmued LC, Stowers CC, Scallet AC, Xu L. Fluoro-Jade C results in ultra high resolution and contrast labeling of degenerating neurons. *Brain Research.* 2005;1035(1):24-31.
3. Ikenari T, Kurata H, Satoh T, Hata Y, Mori T. Evaluation of Fluoro-Jade C Staining: Specificity and Application to Damaged Immature Neuronal Cells in the Normal and Injured Mouse Brain. *Neuroscience.* 2020;425:146-156.
4. Boorman DC, Keay KA. Sex differences in morphine sensitivity are associated with differential glial expression in the brainstem of rats with neuropathic pain. *J Neurosci Res.* 2022;100(10):1890-1907.
5. Brooks SP, Trueman RC, Dunnett SB. Assessment of motor coordination and balance in mice using the rotarod, elevated bridge, and footprint tests. *Current protocols in mouse biology.* 2012;2(1):37-53.
6. Guyenet SJ, Furrer SA, Damian VM, Baughan TD, La Spada AR, Garden GA. A simple composite phenotype scoring system for evaluating mouse models of cerebellar ataxia. *Journal of visualized experiments : JoVE.* 2010(39).
7. Absalom NL, Liao VWY, Kothur K, et al. Gain-of-function GABRB3 variants identified in vigabatrin-hypersensitive epileptic encephalopathies. *Brain Commun.* 2020;2(2):fcaa162.
8. Liao VWY, Chua HC, Kowal NM, Chebib M, Balle T, Ahring PK. Concatenated gamma-aminobutyric acid type A receptors revisited: Finding order in chaos. *J Gen Physiol.* 2019;151(6):798-819.
9. Lin SXN, Ahring PK, Keramidas A, et al. Correlations of receptor desensitization of gain-of-function GABRB3 variants with clinical severity. *Brain.* 2024;147(1):224-239.
10. Yao Z, van Velthoven CTJ, Nguyen TN, et al. A taxonomy of transcriptomic cell types across the isocortex and hippocampal formation. *Cell.* 2021;184(12):3222-3241 e3226.
11. Bryson A, Berkovic SF, Petrou S, Grayden DB. State transitions through inhibitory interneurons in a cortical network model. *PLoS Comput Biol.* 2021;17(10):e1009521.
12. Dos Santos AB, Larsen SD, Gomez CD, Sørensen JB, Perrier J-F. Protocol for quantifying pyramidal neuron hyperexcitability in a mouse model of neurodevelopmental encephalopathy. *STAR protocols.* 2024;5(2):102954.
13. Dura-Bernal S, Suter BA, Gleeson P, et al. NetPyNE, a tool for data-driven multiscale modeling of brain circuits. *Elife.* 2019;8.
14. Hines ML, Davison AP, Muller E. NEURON and Python. *Front Neuroinform.* 2009;3:1.

Supplementary Tables and Figures

Supplementary Table 1. Oligonucleotides for creation of *GABRB3* mouse models and genotyping primers and restriction enzymes.

**Guide sRNAs for Cas9-mediated DNA cleavage**	
β3^{E77K}	CATGGTTTCTGAAGTCAACATGG
β3^{I306T}	GCCCATCAGGTACATGTCGATGG
β3^{L256Q}	CCCACGAGAGGATTGTGATCAGG
β3^{T287I}	TGAGTGTTGATGGTTGTCATGG
**Repair Oligonucleotides (coding regions formatted as codons**	
β3^{E77K}	GT CCC CCA GTC TGC GTG GGG ATG AAC ATC GAC ATC GCC AGC ATC GAC ATG GTT TCG AAG GTC AAC ATG GTGAGTGCCTGCGCTTCCCCAAGCTG TCCCAGGCCCTATCCGGCCGCCTGGGCCCATAC
β3^{I306T}	CTC ACC ATG ACA ACC ATC AAC ACT CAC CTT CGG GAG ACT CTA CCC AAA ATT CCC TAT GTC AAA GCT ACA GAC ATG TAC CTG ATG GGC TGC TTT GTC TTT GTA TTC CTG GCA CTT CTG GAG TAC GCC TTT GTC AA
β3^{L256Q}	A TTG AGT TTT CGG TTG AAG AGA AAT ATC GGG TAC TTC ATT CTT CAG ACG TAT ATG CCC TCA ATC CAA ATC ACC ATC CTC TCG TGG GTG TCC TTC TGG ATC AAT TAC GAT GCA TCT GCT GCT CGA GTT GCC CTT G GTAT
β3^{T287I}	GTGATCTGAGCTGCGGTACTCATACTGCCTTTGCATTTTA GGG ATT ACC ACC GTG CTC ACC ATG ATC ACC ATC AAC ACT CAC CTT CGG GAG ACT CTA CCC AAA ATT CCC TAT GTC AAA GCC ATC GAC ATG TA
**PCR primers and restriction enzymes used for genotyping**	
β3^{E77K}	5'-ATTCGCCTGAGACCCGACTT-3', 5'-TTTCACCTGGGGATGCAAGC-3', BStB1, wild-type allele 660bp, β3^{E77K} allele 383 and 277bp
β3^{I306T}	5'-TCCAGTGGCATTCAGTCCAGT-3', 5'-GTGGAGAGCCTACCCGGTTT-3', SfcI, wild-type allele 360bp, β3^{I306T} allele 181 and 179bp
β3^{L256Q}	5'-TGGGTGGCTCCCCAGTTTATG-3', TTCCTAGTTGCTCTCTGGCCTTT-3', AseI or HphI, wild-type allele 355 bp, β3^{L256Q} allele 191 and 164 bp.
β3^{T287I}	5'-GGCTGGTTAGGGCCTCTGTG-3', 5'-TGGTTGGAGTGTCCAGGCAG-3', BclI, wild-type allele 472 bp, β3^{T287I} allele 255 bp and 217 bp.

Supplementary Table 2. Biophysical properties and reported clinical features of GOF *GABRB3* variants for which mouse models were attempted to be created.

	**p.Glu77Lys**	**p.Ile306Thr**	**p.Leu256Gln**	**p.Thr287Ile**
**Biophysical characteristics**				
Function	GOF	GOF	GOF	GOF
Location	Extracellular Coupling	M2-M3<br>Coupling	Transmembrane (M1)	Transmembrane Pore (M2)
$\Delta LogEC_{50}$	0.64	0.38	0.47	0.52
**Clinical Details**				
No. of Patients	2[1,2]	1[1]	2[1]	2[1]
Age of Onset (m)	4, 192	Unknown	0.03, 1.63	3, 3
Intellectual Disability	Severe, Moderate	Unknown	Severe, Severe	Severe, Severe
	Hypotonia, hypersensitivity to vigabatrin;<br>Trunk dystonia, cerebellum syndrome		Hypotonia, choreoathethosis, non-verbal, nystagmus, tube fed;<br>Hypotonia, dyskinesia	Hypotonia, hypersensitivity to vigabatrin; Involuntary movement, microcephaly
**Living F1 mice[a]**				
Wild-type	53	15	8	29
Knock-in	10	0	0	0
**Dead F1 mice[a]**				
Wild-type	0	3	6	6
Knock-in	0	1	6	0
Unknown[+]	8	3	1	17

[1]Absalom et al, 2022'
[2]Malliard et al, 2022
[a]Prior to weaning
[+]Unknown indicates pups that died before genotyping could be completed.

Supplementary Table 3. Electrophysiological parameters of mIPSC and AP recordings from $\beta 3^{E77K}$-6J CA1 hippocampal neurons and cortical layer 2/3 neurons. All data presented here are mean ± SD.

**Cortical layer 2/3 pyramidal neurons**			
**Parameter**	**WT (n = 14)**	**E77K (n = 15)**	***P*-value**
Averaged peak amplitude (pA)	56.31 ± 12.08	39.75 ± 14.22	0.002
Area	463.40 ± 78.16	354.80 ± 137.72	0.02
Frequency (Hz)	1.41 ± 0.62	3.03 ± 1.12	<0.0001
Rise time (ms)	0.49 ± 0.09	0.86 ± 0.30	0.0001
Half width (ms)	4.12 ± 0.53	4.93 ± 1.17	0.004
Decay time (ms)	2.07 ± 0.39	2.87 ± 0.52	<0.0001
Membrane capacitance (pF)	173.80 ± 43	182.80 ± 40.97	0.57
**Parameter**	**WT (n = 10)**	**E77K (n = 6)**	**P-value**
F-I plot			0.02
Rm at rest (MΩ)	-74.55 ± 4.14	-77.83 ± 2.91	0.11
Input resistance (MΩ)	98.30 ± 38.85	57.21 ± 17.64	0.03
Rheobase (pA)	144 ± 80.44	253.30 ± 58.88	0.01
Membrane capacitance (pF)	188.80 ± 52	206.70 ± 35.70	0.47
**Cortical layer 2/3 interneurons**			
**Parameter**	**WT (n = 16)**	**E77K (n = 15)**	***P*-value**
Averaged peak amplitude (pA)	29.21 ± 6.91	46.57 ± 9.79	<0.0001
Area	266.7 ± 41.17	440.7 ± 69.77	<0.0001
Frequency (Hz)	1.53 ± 0.94	2.08 ± 1.99	0.32
Rise time (ms)	1.17 ± 0.24	1.13 ± 0.46	0.80
Half width (ms)	4.68 ± 0.82	4.83 ± 0.97	0.65
Decay time (ms)	2.64 ± 0.41	3.07 ± 0.55	0.02
Membrane capacitance (pF)	48.53 ± 12.40	50.58 ± 7.52	0.59
**Parameter**	**WT (n = 5)**	**E77K (n = 6)**	**P-value**
F-I plot			0.66
Rm at rest (MΩ)	-70.70 ± 3.48	-71.84 ± 5.27	0.69
Input resistance (MΩ)	102.50 ± 24.62	108.80 ± 44.39	0.78
Rheobase (pA)	180 ± 73.48	216.70 ± 88.92	0.48
Membrane capacitance (pF)	46.11 ± 13.80	51.14 ± 8.57	0.44

**CA1 Hippocampal pyramidal neurons**			
**Parameter**	**WT (n = 12)**	**E77K (n = 16)**	***P*-value**
Averaged peak amplitude (pA)	35.96 ± 10.64	52.05 ± 10.16	0.0004
Area	499.40 ± 159	715.20 ± 255.52	0.02
Frequency (Hz)	5.77 ± 2.80	2.89 ± 1.67	0.002
Rise time (ms)	0.92 ± 0.17	0.63 ± 0.16	<0.0001
Half width (ms)	5.79 ± 1.10	4.60 ± 0.63	0.001
Decay time (ms)	3.66 ± 0.74	2.53 ± 0.50	<0.0001
**Parameter**	**WT (n = 11)**	**E77K (n = 16)**	**P-value**
F-I plot			0.97
Rm at rest (MΩ)	-65.17 ± 3.46	-64.10 ± 2.97	0.44

Input resistance (MΩ)	167.1 ± 58.64	174.90 ± 55.49	0.75
Rheobase (pA)	55.45 ± 33.86	42.50 ± 25.98	0.31
**CA1 Hippocampal interneurons**			
**Parameter**	**WT (n = 11)**	**E77K (n = 12)**	***P*-value**
Averaged peak amplitude (pA)	39.83 ± 14.58	57.11 ± 16.27	0.01
Area	258 ± 80.39	479 ± 205.70	0.003
Frequency (Hz)	4.77 ± 1.83	2.57 ± 1.77	0.008
Rise time (ms)	0.93 ± 0.19	0.73 ± 0.28	0.047
Half width (ms)	4.20 ± 0.53	4.86 ± 0.99	0.047
Decay time (ms)	2.53 ± 0.34	3.17 ± 1.01	0.06

Supplementary Table 4. Electrophysiological parameters of TEVC recordings from *Xenopus* oocytes expressing the single $\beta3^{E77K}$ variant, double $\beta3^{E77K}$, or WT concatenated $GABA_A$ receptor (γ2-β3-α1-β3-α1). All data presented here are mean ± SD unless otherwise specified.

	**EC_{50} ± SD (µM)**	**$LogEC_{50}$ ± SD**	**$\Delta LogEC_{50}$ ± SD**	**nH ± SD**	**n**	***P* ($\Delta LogEC_{50}$)[a]**
**WT**	310 ± 72	-3.5 ± 0.11	0.00 ± 0.10	1.4 ± 0.11	13	NA
**Single E77K**	87 ± 10	-4.1 ± 0.05	0.54 ± 0.06	1.3 ± 0.08	11	< 0.0001
**Double E77K**	62 ± 10	-4.2 ± 0.08	0.69 ± 0.08	1.0 ± 0.08	12	< 0.0001

[a]One-way ANOVA with Dunnett's post-hoc test

	**Normalised I_{max} median [IQR]**	**n**	***P* (I_{max})[b]**
**WT**	1.00 [0.60-2.07]	24	NA
**Single E77K**	0.68 [0.44-0.89]	24	0.0054
**Double E77K**	0.13 [0.085-0.20]	24	< 0.0001

[b]Mann-Whitney U test

	**k (s^{-1})**	**n**	**P(k)*** **	**I_{ss}/I_{peak}**	**n**	**P(I_{ss}/I_{peak})[c]**
**WT**	0.01 ± 0.002	26	NA	0.22 ± 0.04	26	NA
**Single E77K**	0.02 ± 0.005	15	0.02	0.21 ± 0.07	15	0.39
**Double E77K**	0.02 ± 0.005	14	< 0.0001	0.17 ± 0.05	14	0.007

[c]Non-parametric one-way ANOVA (Kruskal-Wallis rank sum test) with Dunnett's post-hoc test.

Supplementary Table 5. Behavioural assays and parameters conducted to characterise the $\beta3^{E77K}$-6J mouse model. All data presented here are mean ± SD. n numbers are also reported here. Unless otherwise stated, data were analysed using an unpaired two-tailed student's *t*-test.

**Locomotor/Thigmotaxis (P21-34)**			
**Parameter**	**WT (n = 15)**	**$\beta3^{E77K}$ (n = 13)**	***P*-value**
Distance travelled (m)	36.37 ± 11.98	30.50 ± 11.04	0.26[a]
Vertical jump count	79 ± 47.45	25.92 ± 36.62	0.0008 [a]
Average speed (mm/s)	32.55 ± 5.289	29.01 ± 4.286	0.09 [a]
Distance travelled in centre zone (% of total)	41.63 ± 7.87	41.47 ± 6.54	0.96
**Locomotor/Thigmotaxis (P45-60)**			
**Parameter**	**WT (n = 9)**	**$\beta3^{E77K}$ (n = 9)**	***P*-value**
Distance travelled (m)	86.39 ± 16.87	43.66 ± 17.49	<0.0001
Resting time (s)	2319 ± 136.20	2810 ± 271.70	0.0002
Vertical jump count	510 ± 144.40	196.7 ± 141.50	0.0003
Average speed (mm/s)	33.30 ± 3.28	25.93 ± 3.71	0.0004
Distance travelled in centre zone (% of total)	60.31 ± 7.84	44.23 ± 6.48	0.0008 [a]
**Rotarod**			
**Parameter**	**WT (n =10)**	**$\beta3^{E77K}$ (n = 9)**	***P*-value**
Run 1 (s)	184 ± 61.54	176.90 ± 59.91	0.80
Run 2 (s)	252.40 ± 54.77	224.10 ± 45.15	0.24
Run 3 (s)	236.40 ± 61.94	214.40 ± 35.34	0.36
Combined average over all 3 trials (s)	224.30 ± 53.16	205.10 ± 43.89	0.41
**Ledged Beam**			
**Parameter**	**WT (n =10)**	**$\beta3^{E77K}$ (n = 10)**	***P*-value**
Foot faults	2.80 ± 1.69	3.90 ± 3.04	0.8631 [a]
Time on beam (s)	0.06 ± 0.04	0.09 ± 0.06	0.6939 [a]
**Grip Strength**			
**Parameter**	**WT (n =11)**	**$\beta3^{E77K}$ (n = 11)**	***P*-value**
Average force (g)	89.94 ± 23.12	68.72 ± 7.24	0.009
**Clasping**			
**Parameter**	**WT (n = 11)**	**$\beta3^{E77K}$ (n = 11)**	***P*-value**
Hindlimb clasping score	0.00 ± 0.00	0.10 ± 0.30	>0.9999 [a]
**Light-Dark Test**			
**Parameter**	**WT (n = 10)**	**$\beta3^{E77K}$ (n = 9)**	***P*-value**
Duration in light zone (%)	29.33 ± 10.82	42.60 ± 8.79	0.01
Distance travelled in light zone (% of total)	32.07 ± 7.76	40.05 ± 4.70	0.016
Latency to enter light zone (s)	72.96 ± 94.46	49.86 ± 40.92	0.80 [a]
**Elevated Plus Maze**			
**Parameter**	**WT (n = 10)**	**$\beta3^{E77K}$ (n = 9)**	***P*-value**
Duration in open arm (%)	10.75 ± 4.97	9.50 ± 4.61	0.59
Bouts in open arm	10.50 ± 4.88	9.78 ± 4.38	0.74

Three-chamber social interaction			
**Parameter**	**WT (n = 10)**	**β3^{E77K} (n = 10)**	***P*-value**
Duration interaction zone (%)	23.82 ± 6.29	26.30 ± 5.00	0.34
Duration centre zone (%)	25.40 ± 11.39	23.19 ± 5.815	0.59
Duration avoidance zone (%)	15.91 ± 3.56	13.92 ± 3.25	0.21
**Y-maze**			
**Parameter**	**WT (n = 9)**	**β3^{E77K} (n = 8)**	***P*-value**
Duration in novel arm (%)	33.58 ± 6.74	28.14 ± 5.02	0.08
Novel arm bouts	11.11 ± 2.83	9.13 ± 2.30	0.14
**Barnes maze**			
**Parameter**	**WT (n = 10)**	**β3^{E77K} (n = 10)**	***P*-value**
Day 1 latency (s)	40.94 ± 19.83	36.32 ± 12.83	0.54
Day 2 latency (s)	40.66 ± 18.46	29.98 ± 17.39	0.20
Day 3 latency (s)	29.13 ± 16.01	22.79 ± 13.79	0.35
Day 4 latency (s)	30.41 ± 13.44	19.28 ± 5.587	0.03
Day 5 latency (s)	28.61 ± 22.97	18.38 ± 9.854	0.21
Reversal latency	80.88 ± 48.10	71.28 ± 51.42	0.67
Probe test latency day 10	26.81 ± 19.32	20.16 ± 19.32	0.48

[a] Data were analysed using a Mann-Whitney test.

Supplementary Table 6. Statistical comparison of $GABA_A$ receptor subunit mRNA expression in PV^+ interneurons vs pyramidal neurons.

	**p_val**	**avg_log2FC**	**pct.1**	**pct.2**	**p_val_adj**
**Gabrd**	7.79E-80	-2.06	0.66	0.91	3.28E-75
**Gabrb3**	8.02E-72	-1.22	0.99	1	3.37E-67
**Gabra4**	2.29E-64	-1.42	0.88	0.96	9.61E-60
**Gabra2**	3.66E-64	-1.72	0.63	0.88	1.54E-59
**Gabrg2**	3.69E-58	-1.04	0.98	0.99	1.55E-53
**Gabrb2**	1.68E-51	-0.87	0.98	0.99	7.05E-47
**Gabrb1**	4.33E-51	-0.98	0.98	0.99	1.82E-46
**Gabra1**	1.68E-48	-0.828	0.97	0.99	7.08E-44
**Gabra3**	1.54E-09	-0.69	0.74	0.79	6.47E-05
**Gabrg3**	1.85E-09	0.26	0.95	0.79	7.78E-05
**Gabrp**	0.004	-0.43	0.07	0.12	1
**Gabrg1**	0.007	-1.11	0.12	0.17	1

Supplementary Table 7. Behavioural assays and parameters conducted to characterise the $\beta3^{E77K}$-6N mouse model. All data presented here are mean ± SD. n numbers are also reported here. Unless otherwise stated, data were analysed using an unpaired two-tailed student's *t*-test.

**Locomotor/Thigmotaxis (P40)**			
**Parameter**	**WT (n = 10)**	**$\beta3^{E77K}$ (n = 10)**	***P*-value**
Distance travelled (m)	43.02 ± 15.17	39.88 ± 15.70	0.35*
Vertical jump count	209.60 ± 53.80	156.10 ± 54.69	0.04
Average speed (mm/s)	29.50 ± 4.18	31.55 ± 3.50	0.25
Distance travelled in centre zone (% of total)	40.66 ± 7.42	41.62 ± 8.90	0.80
**Clasping**			
**Parameter**	**WT (n = 9)**	**$\beta3^{E77K}$ (n = 13)**	***P*-value**
Hindlimb clasping score	0.20 ± 0.30	1.30 ± 0.72	<0.0001*

Supplementary Figure 1

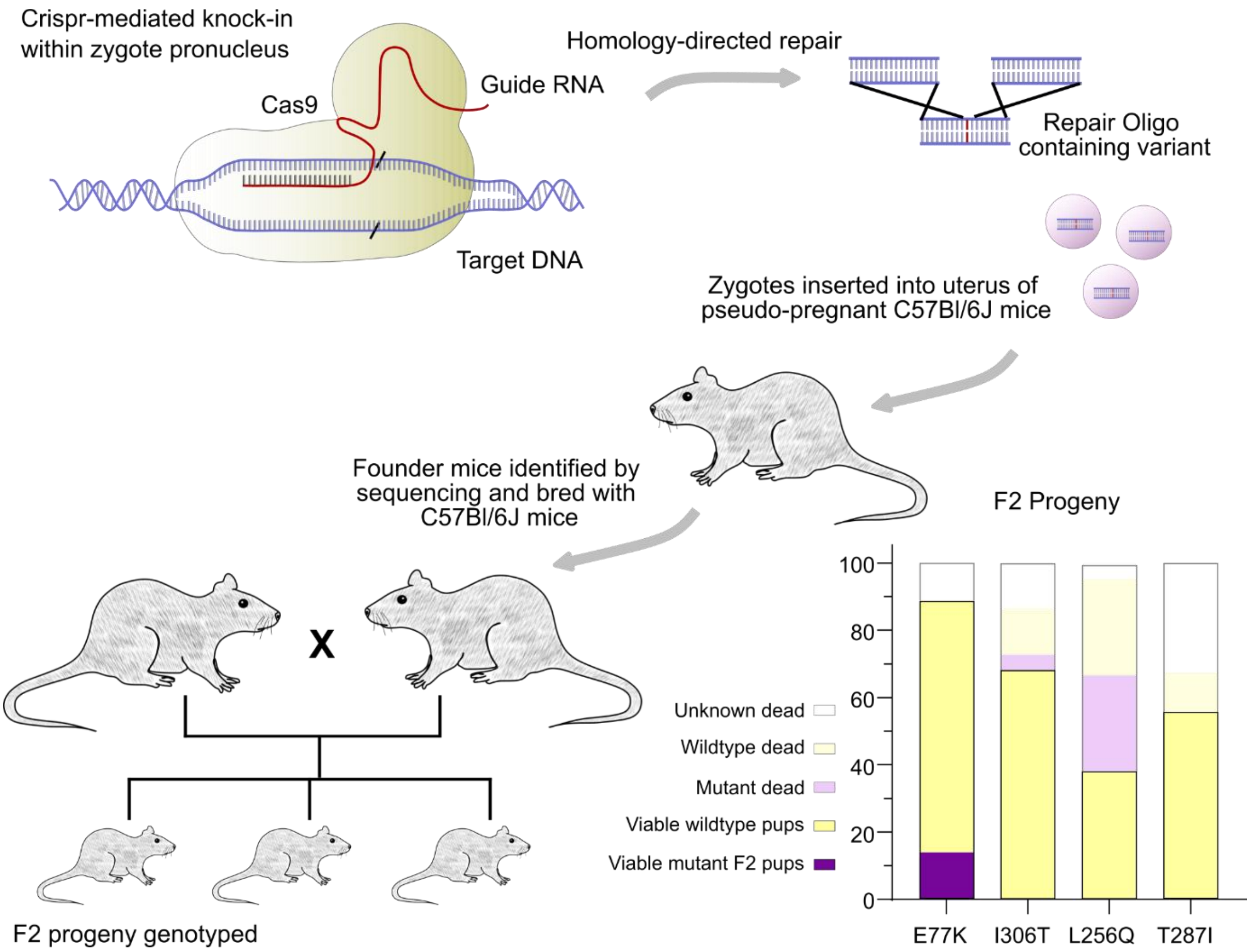

Supplementary Figure 1. Generation of the β3^{E77K} mouse model.

Supplementary Figure 2

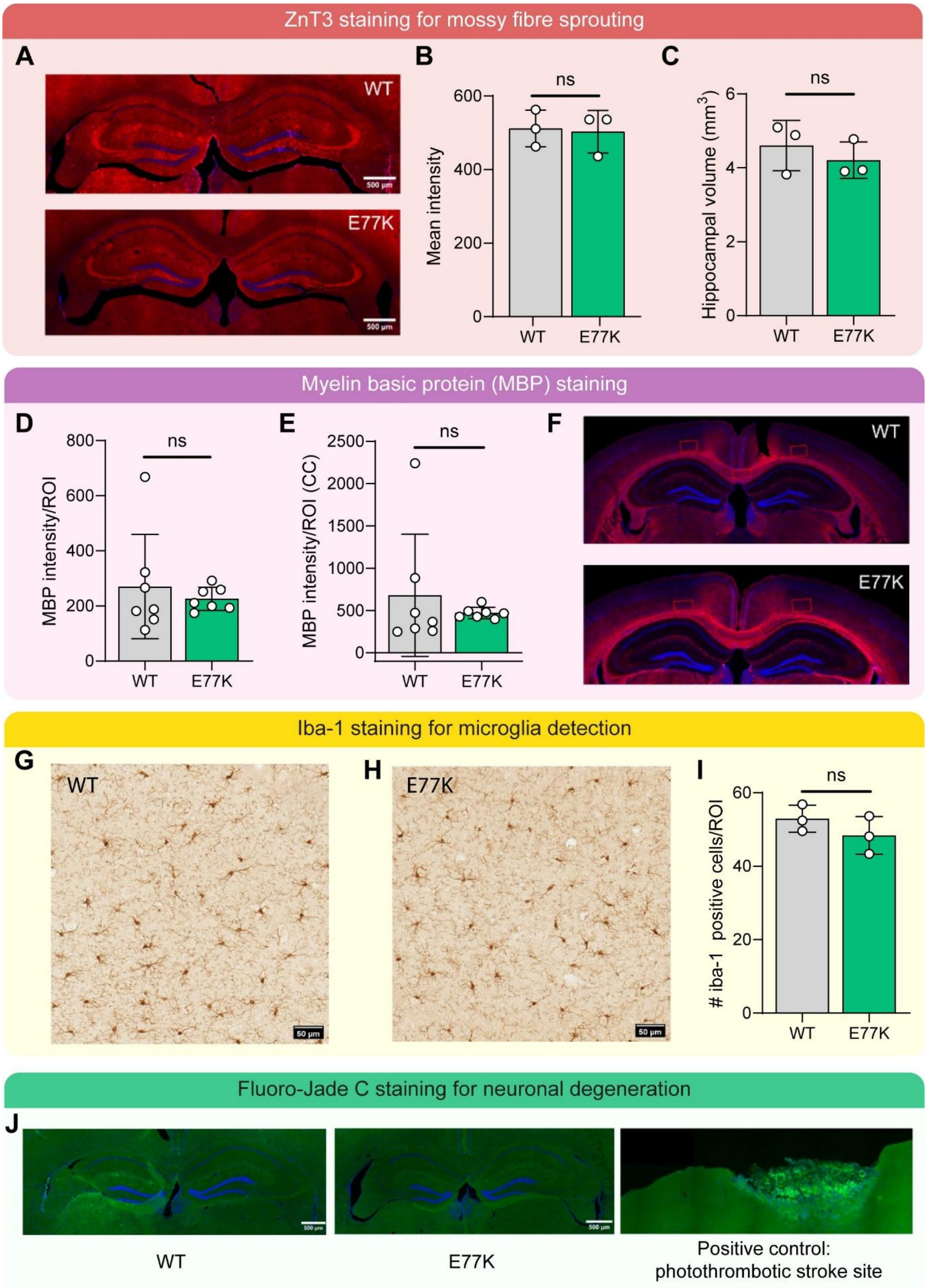

Supplementary Figure 2. β3^{E77K}-6J mice displayed no significant changes in morphological markers of excitability. **(A)** Representative image of ZnT3 immunohistochemistry staining for mossy fibre sprouting for WT (*top*, n = 3 mice) and E77K (*bottom*, n = 3 mice). Scale bar = 500 µm. **(B)** Bar

graph depicting the mean intensity of ZnT3 for WT and E77K, $P > 0.9999$, Mann-Whitney test. **(C)** There was no difference in total hippocampal volume (n = 3 WT, 3 β3^{E77K}), $P = 0.9999$, unpaired *t*-test. **(D)** Bar graph of average fluorescence intensity per region of interest, $P = 0.8048$, Mann-Whitney test. **(E)** Bar graph of mean intensity per region of interest (corpus callosum), $P = 0.4557$, Mann-Whitney test. **(F)** Representative photograph of myelin basic protein in WT (*top*) and E77K (*bottom*). **(G)** and **(H)** Representative Iba-1 immunostaining for WT (*left*) and E7K (*right*). Scale bar = 50 µm **(I)** Bar graph of Iba-1 positive cells per region of interest (n = 3 per group for WT and E77K, $P = 0.7$, Mann-Whitney test). **(J)** Representative photograph of FJC staining for WT (*left*) E77K (*middle*) and (*right*) a tissue from a mouse that previously underwent photothrombotic stroke as a positive control. Scale bars are 500 µm for WT and E77K. Data presented as mean ± SD.

Supplementary Figure 3

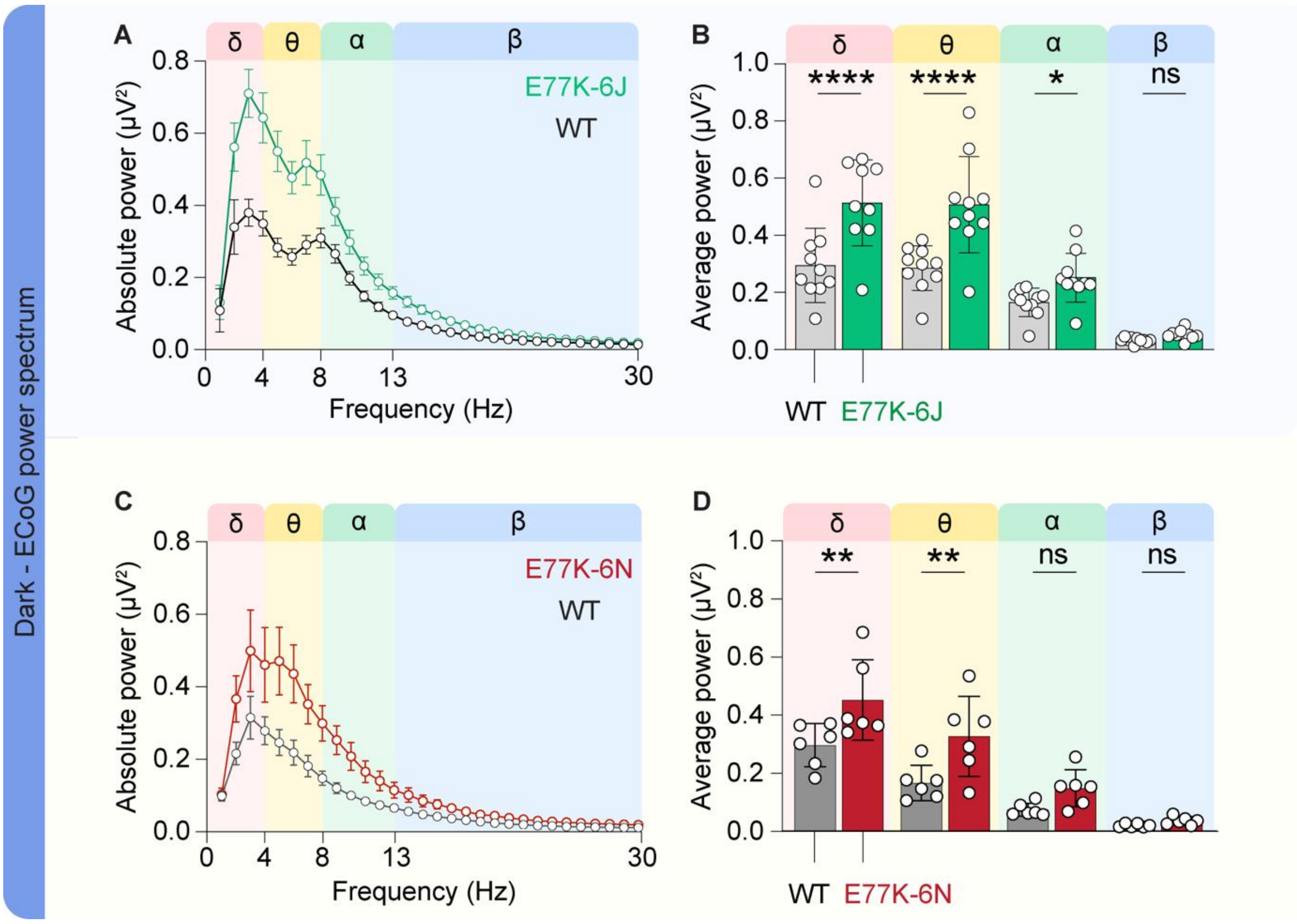

Supplementary Figure 3. β3^{E77K} mice displayed increased absolute ECoG power in the dark phase. (A) Absolute spectral power. **(B)** Mean absolute spectral power in β3^{E77K}-6J mice (n = 10 per group) was significantly increased compared with WT across all frequency bands in the dark phase. Two-way repeated-measures ANOVA showed a significant main effect of genotype ($F(3,36) = 28.54$, $P < 0.0001$), frequency band ($F(1,36) = 71.00$, $P < 0.0001$) and a significant band x genotype interaction ($F(3,36) = 11.07$, $P < 0.0001$). Holm-Šidák post-hoc tests confirmed significantly increased delta (adjusted $P < 0.0001$), theta (adjusted $P < 0.0001$), and alpha power (adjusted $P = 0.0428$) in β3^{E77K} mice, with no significant difference in beta power (adjusted $P = 0.5988$). **(C)** Absolute spectral power. **(D)** β3^{E77K}-6N mice (n = 6 per group) had significantly increased ECoG spectral power across power bands in the dark phase relative to WT. Mean absolute spectral power in β3^{E77K}-6N mice (n = 6 per group) was significantly increased compared with WT. Two-way repeated-measures ANOVA identified a significant main effect of genotype ($F(1,20) = 41.38$, $P < 0.0001$) and frequency band ($F(1,20) = 19.29$, $P = 0.0003$), with no significant interaction ($F(3,20) = 2.28$, $P = 0.11$). Holm-Šidák post-hoc tests confirmed significantly elevated delta (adjusted

$P = 0.0094$) and theta power (adjusted $P = 0.0094$) in β3^{E77K} mice, with no significant difference in alpha or beta bands (adjusted $P = 0.2376$ and $P = 0.7361$, respectively). Data presented as mean ± SEM for power spectrum and mean ± SD for average power graphs.

Supplementary Figure 4

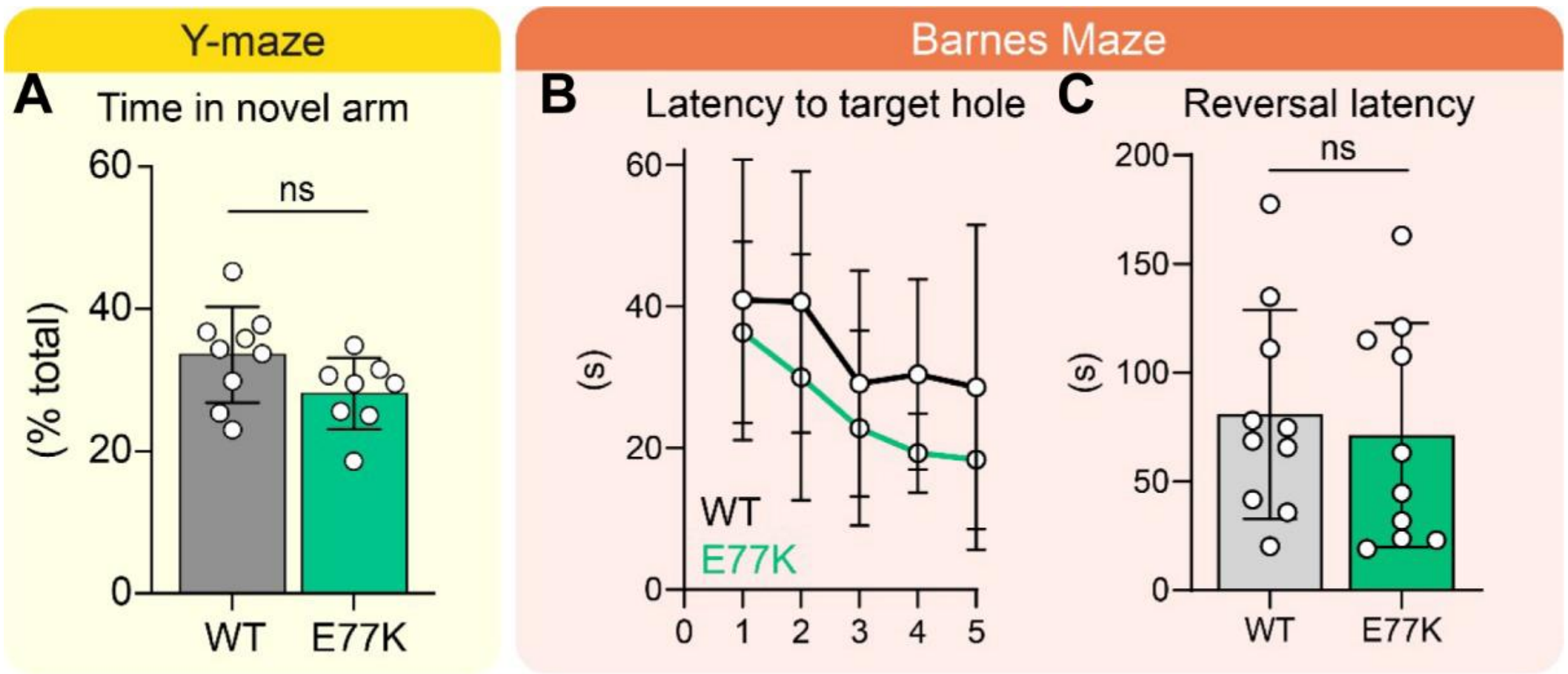

Supplementary Figure 4. β3^{E77K}-6J mice displayed no significant changes in learning and memory. (A) The percentage of time spent in the novel arm of the Y-maze was not significantly different between genotypes (n = 9 WT, 8 β3^{E77K}), $P = 0.0817$, unpaired *t*-test. **(B)** There were no significant differences between β3^{E77K} mice and wild-type littermates in average latency to target in the Barnes maze assay (n = 10 WT, 10 β3^{E77K}), $P = 0.5444$, $P = 0.1995$, $P = 0.3554$, $P = 0.0265$, $P = 0.2121$, on days 1-5, respectively, multiple *t*-tests. **(C)** Time to reversal latency (during which the target hole is reversed to the opposite side of the maze, performed on day 10 of the test) was not different between the two groups (n = 10 WT, 10 β3^{E77K}), $P = 0.6716$, unpaired *t*-test. Data presented as mean ± SD.

Supplementary Figure 5

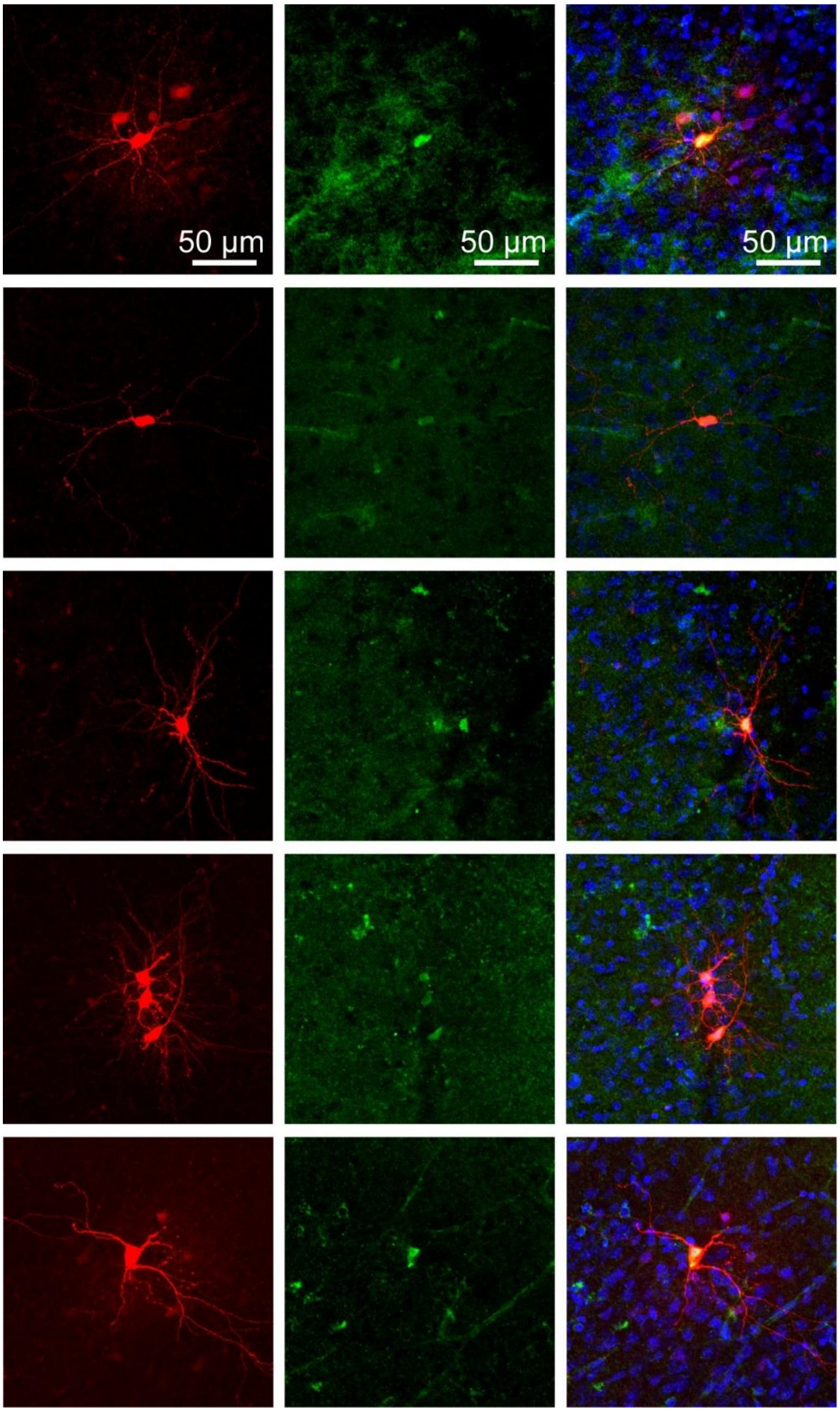

Supplementary Figure 5. Representative confocal images showing the morphology and staining of L2/3 cortical interneurons. Immunofluorescent images of fast-spiking interneurons from $\beta3^{E77K}$-6J mice showing biocytin-filled cells (streptavidin, *red*; *left*), parvalbumin (PV) staining (*green*; *middle*), and merged overlay with DAPI (*blue*; *right*).

Supplementary Figure 6

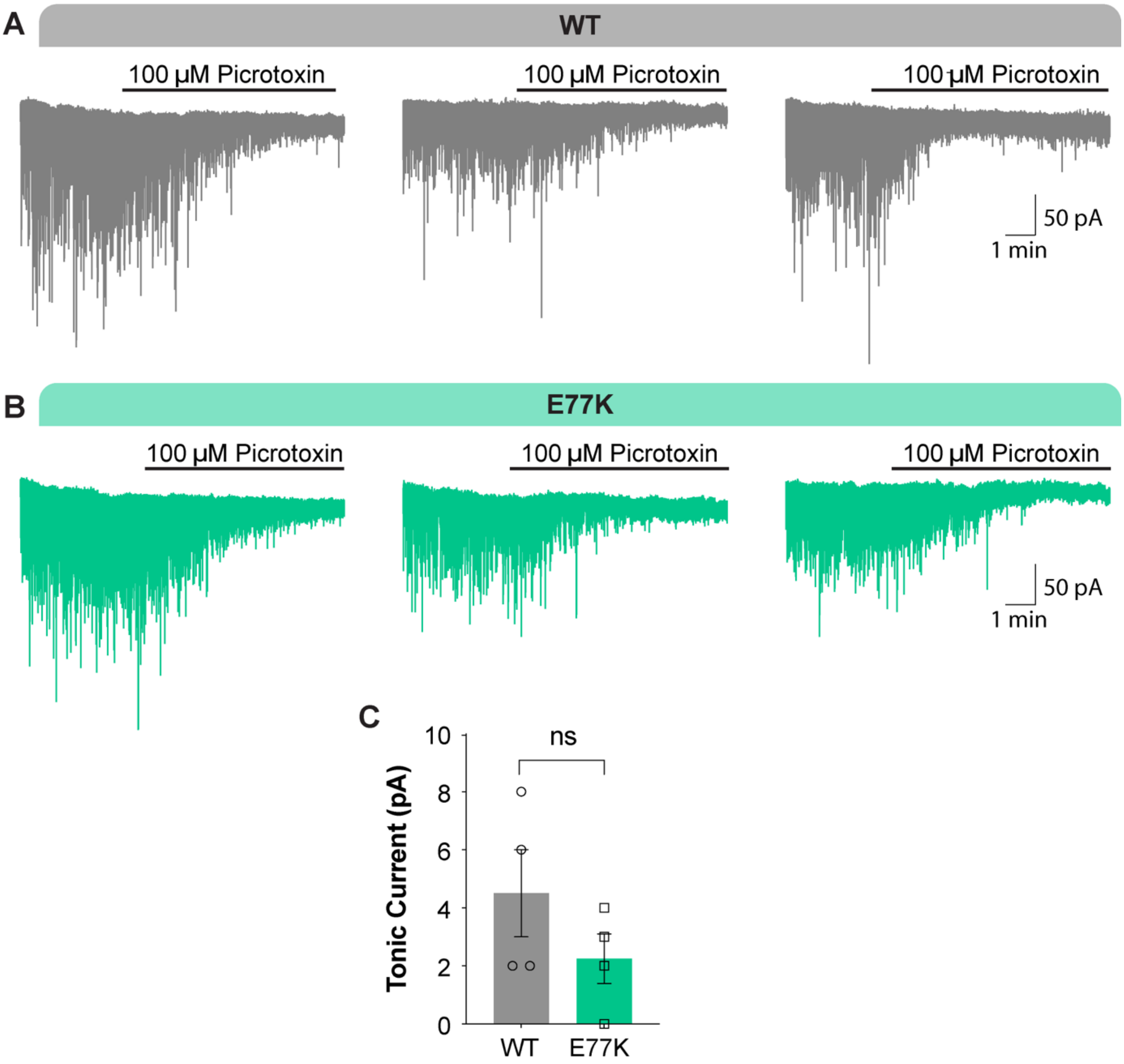

Supplementary Figure 6. Tonic GABA$_A$ receptor-mediated currents in L2/3 pyramidal neurons. Representative whole-cell current traces from WT **(A)** and β3^{E77K}-6J **(B)** layer 2/3 neurons before and after bath application of 100 µM picrotoxin. No clear tonic current shift was observed in either genotype. **(C)** Summary of tonic current amplitude in WT (n = 4) and β3^{E77K}-6J (n = 4) neurons; values were not significantly different (unpaired *t*-test, $p > 0.05$).

Supplementary Figure 7

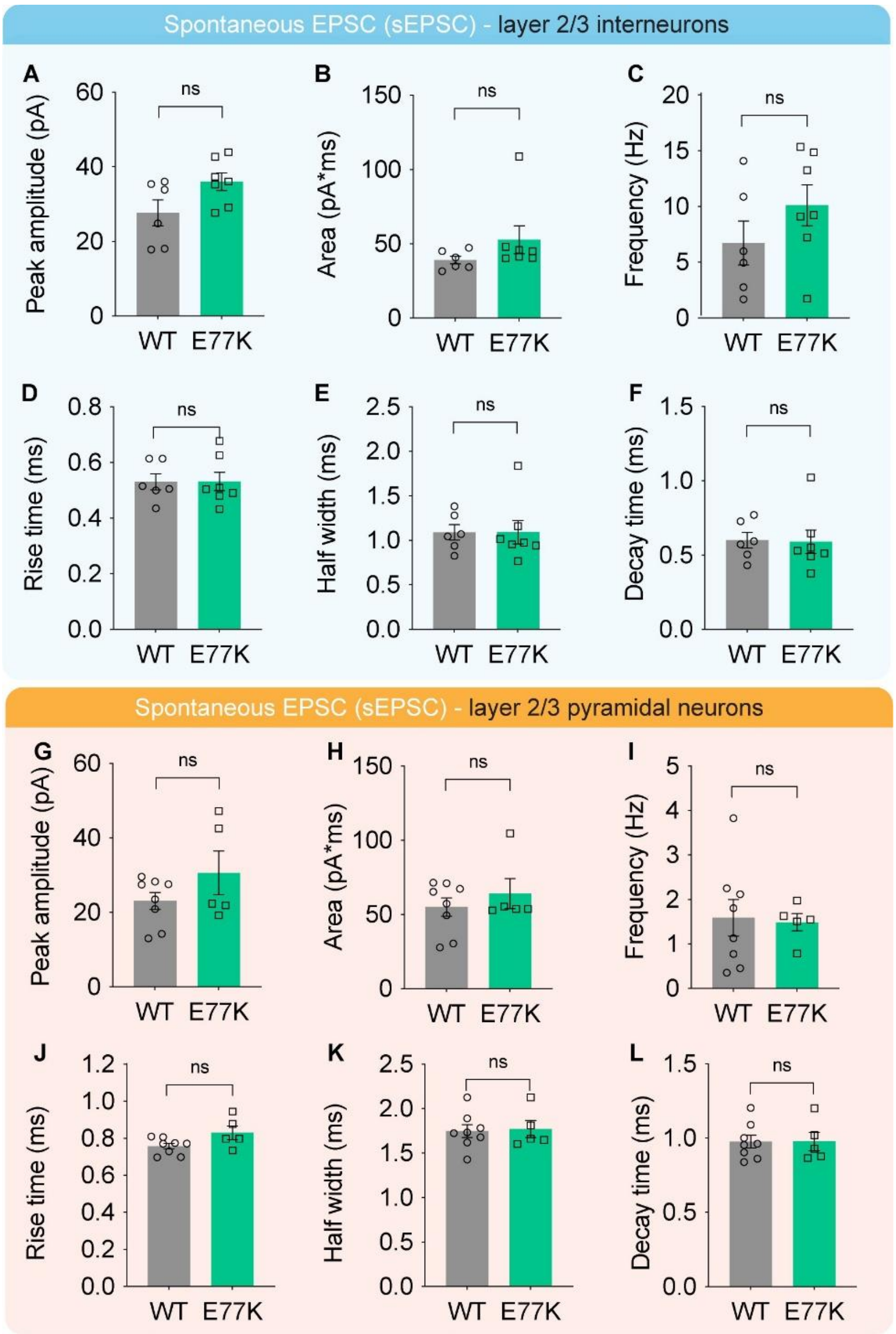

Supplementary Figure 7. Spontaneous excitatory postsynaptic currents (sEPSCs) in β3^{E77K}-6J mice. (A-F) sEPSC properties recorded from layer 2/3 cortical interneurons (WT n = 6, E77K n = 7). **(A)** Peak amplitude (p = 0.0654), **(B)** area under the curve (p = 0.2143), **(C)** frequency (p = 0.2342), **(D)** rise time (p = 0.9837), **(E)** half-width (p = 0.9872), and **(F)** decay time (p = 0.9213); unpaired t-tests for all. **(G-L)** sEPSCs recorded from layer 2/3 cortical pyramidal neurons (WT n = 8, E77K n = 5). **(G)** Peak amplitude (p = 0.1889), **(H)** area under the curve (p = 0.4358), **(I)** frequency (p = 0.8599), **(J)** rise time (p = 0.0574), **(K)** half-width (p = 0.8536), and **(L)** decay time (p = 0.9825); unpaired t-tests for all. No significant differences were observed between genotypes. Data are mean ± SEM.

Supplementary Figure 8

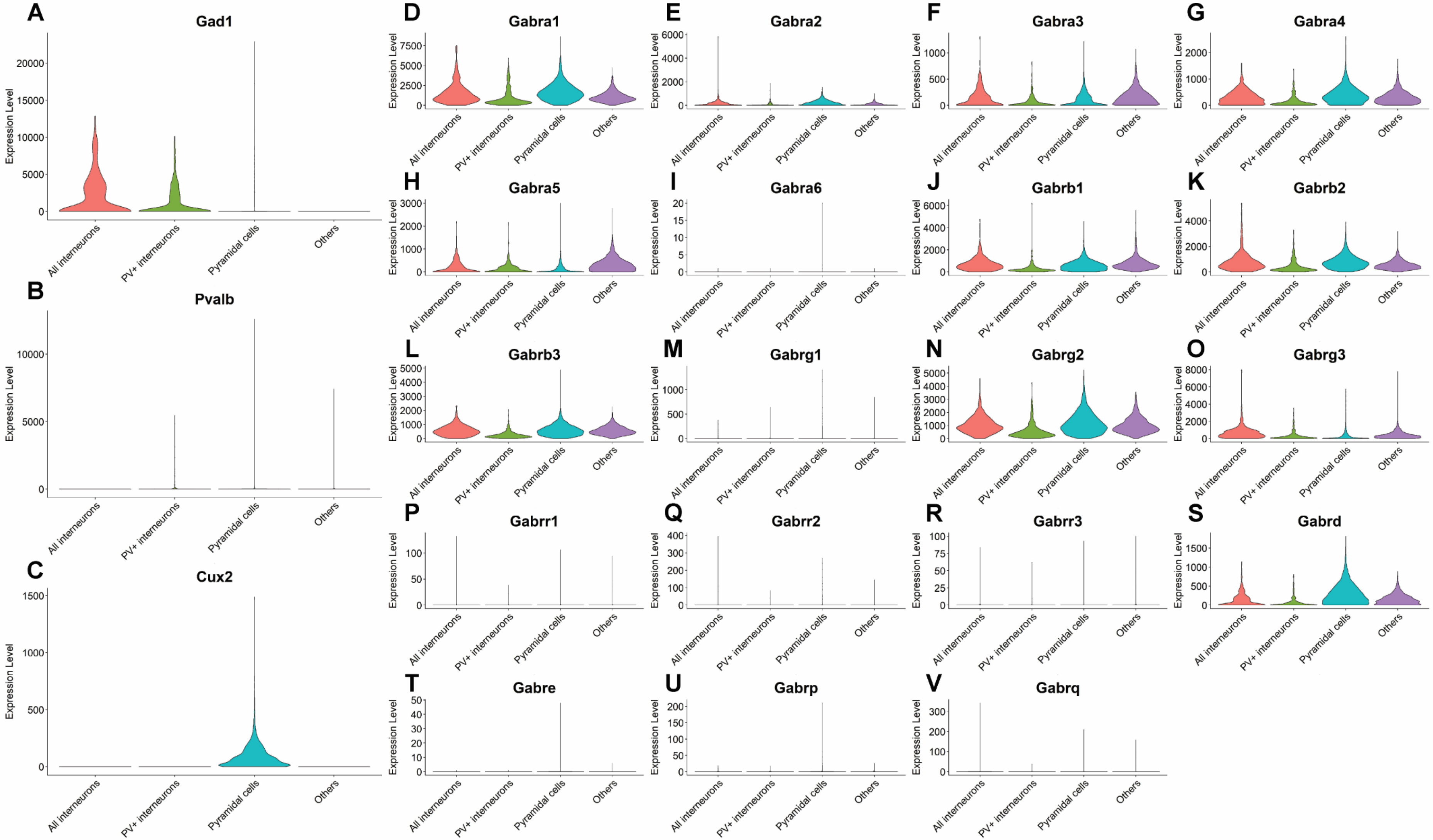

Supplementary Figure 8. Single cell RNA sequencing of mouse whole cortex showing expression of $GABA_A$ receptor genes in all interneurons, PV positive interneurons, pyramidal cells and all other cells. (A-C) Expression of gene markers used to identify different cell types. **(D-V)** Expression of all 19 genes which encode for a $GABA_A$ receptor subunit in each type of cell in the mouse cortex and hippocampus.

Supplementary Figure 9

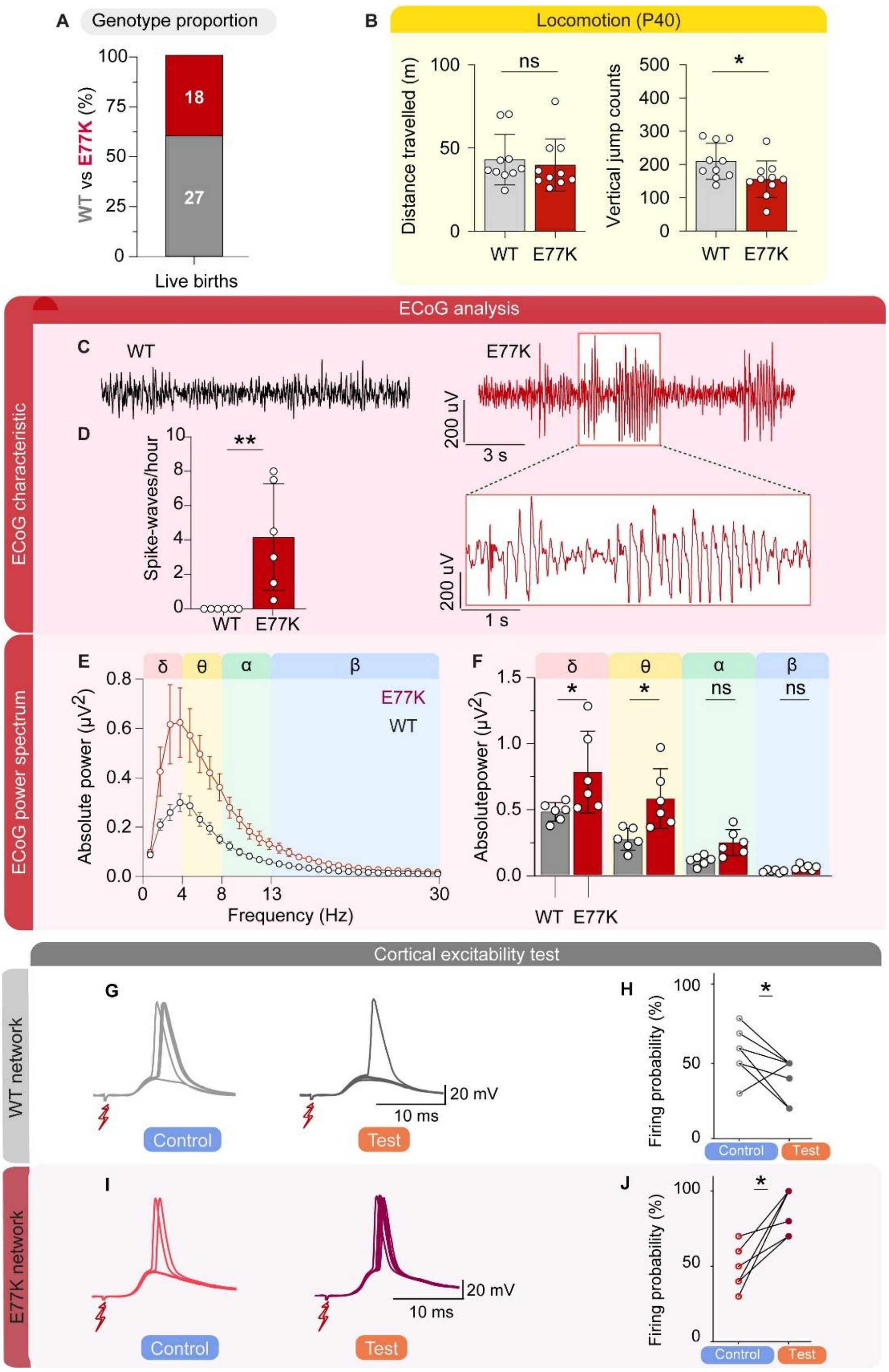

Supplementary Figure 9. $\beta3^{E77K}$-6N mice replicate the epileptic and network hyperexcitability phenotype. (A) Genotype distribution of live births showing a slight reduction in $\beta3^{E77K}$ offspring relative to wild-type (WT) littermates (18 $\beta3^{E77K}$, 27 WT, P = 0.18, Chi-square test). **(B)** Locomotor activity at P 40. Total distance travelled did not differ significantly between genotypes (n = 10/group, P = 0.3527, Mann-Whitney test), whereas vertical jump counts were significantly reduced in $\beta3^{E77K}$ mice (*P = 0.0407, unpaired t-test).

(C) Representative electrocorticography (ECoG) traces from WT (*left*, *black*) and $\beta3^{E77K}$ (*right*, *red*) mice showing recurrent spike-wave discharges (SWDs). The boxed region indicates an individual SWD expanded below. **(D)** Quantification of SWDs per hour revealed a significant increase in $\beta3^{E77K}$ mice compared with WT (n = 6/group, $^{**}P = 0.0022$, Mann-Whitney test). **(E)** ECoG power spectra recorded during the light phase. **(F)** Mean absolute spectral power across the delta (0.5-4 Hz), theta (4-8 Hz), alpha (8-12 Hz), and beta (12-30 Hz) bands recorded during the light phase. $\beta3^{E77K}$ mice exhibited elevated power across all bands relative to WT. Two-way repeated-measures ANOVA identified significant main effects of genotype ($F(3,20) = 55.71$, $P < 0.0001$) and frequency band ($F(1,20) = 15.82$, $P = 0.0007$), with no significant genotype x band interaction ($F(3,20) = 1.94$, $P = 0.1554$). Holm-Šidák post hoc tests confirmed significantly increased delta and theta power in $\beta3^{E77K}$ mice (adjusted $P = 0.0192$ for both), with no significant differences in alpha or beta bands (adjusted $P > 0.05$). n = 6 per group. **(G)** Example current-clamp traces from layer 2/3 pyramidal neurons in WT mice recorded in response to layer 4 stimulation under control and test conditions illustrating decreased firing probability after conditioning. **(H)** Population data showing a significant reduction in action potential firing probability in WT cells (n = 14 trials from 7 cells; $P < 0.05$, Wilcoxon matched-pairs signed rank test). **(I)** Example current-clamp traces from $\beta3^{E77K}$ pyramidal neurons recorded under the same protocol showing increased excitability and loss of inhibitory gating. **(J)** Population data showing significantly increased firing probability in $\beta3^{E77K}$ neurons (n = 12 trials from 6 cells; $P = 0.01$, Wilcoxon matched-pairs signed rank test).

Supplementary Figure 10

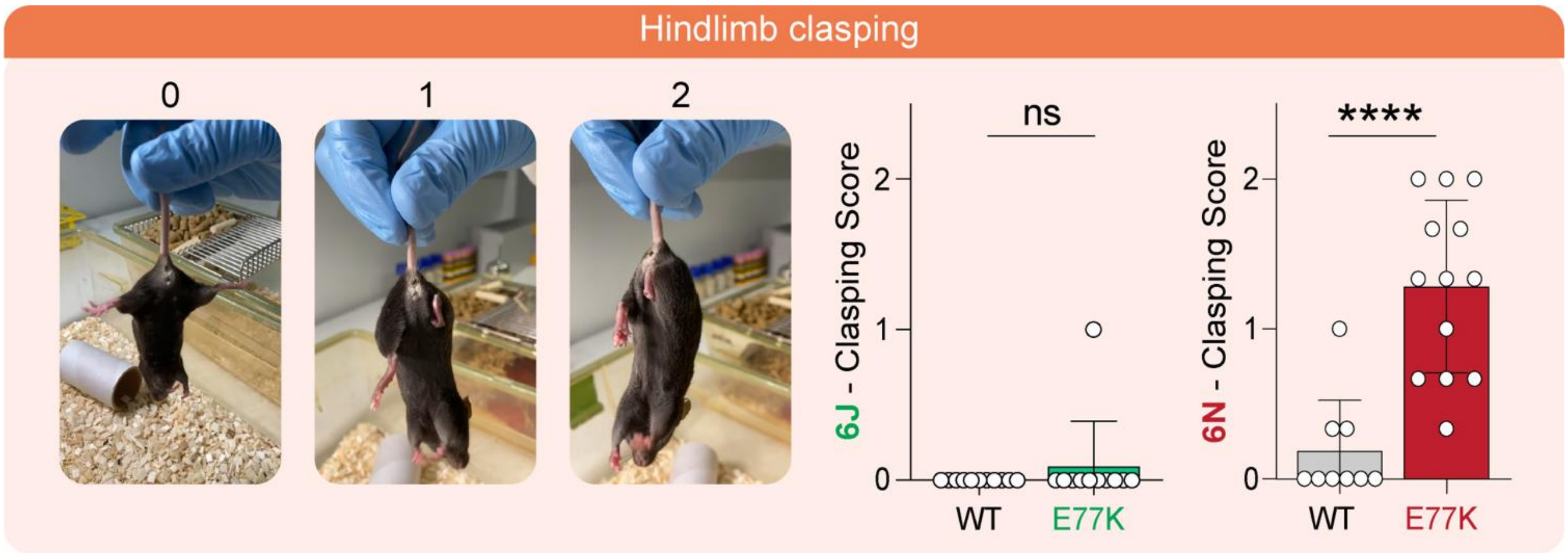

Supplementary Figure 10. **β3^{E77K}-6N, but not β3^{E77K}-6J, mice display increased hindlimb clasping.** The clasping scores of β3^{E77K}-6J animals was not significantly different from WT (n = 11 per group, $P > 0.9999$, Mann-Whitney test). The clasping scores of β3^{E77K}-6N animals were significantly higher than for WT littermates (n = 9 WT, 13 β3^{E77K}), ****$P < 0.0001$, Mann-Whitney test. Representative examples of 0, 1 and 2 clasping scores observed in β3^{E77K}-6N mice.
